# Law and Order of Colloidal Tectonics: From Molecules to Self-Assembled Colloids

**DOI:** 10.3390/molecules29235657

**Published:** 2024-11-29

**Authors:** Loïc Leclercq

**Affiliations:** Univ. Lille, CNRS, Centrale Lille, Univ. Artois, UMR 8181-UCCS, Unité de Catalyse et Chimie du Solide, Lille 59000, France; loic.leclercq@univ-lille.fr

**Keywords:** colloidal tectonics, self-assembly, particles, biologic systems, abiotic systems, catalysis, pharmaceutical applications

## Abstract

Since biochemists and biologists have progressed in understanding the mechanisms involved in living organisms, biological systems have become a source of inspiration for chemists. In this context, the concept of colloidal tectonics, describing the spontaneous formation of colloidal particles or supracolloidal structures in which the building blocks are called “tectons”, has emerged. Therefore, a bottom-up edification of tectons towards (supra) colloidal structures is allowed. Each (supra) colloidal system has at least one of the following properties: amphiphilicity, predictability, versatility, commutability, and reversibility. However, for these systems to perform even more interesting functions, it is necessary for tectons to have very precise chemical and physical properties so that new properties emerge in (supra) colloidal systems. In this way, colloidal tectonics enables engineering at the nano- and micrometric level and contributes to the development of smart bioinspired systems with applications in catalysis, drug delivery, etc. In this review, an overview of the concept of colloidal tectonics is illustrated by some biotic systems. The design of abiotic (supra) colloidal systems and their applications in various fields are also addressed (notably Pickering emulsions for catalysis or drug delivery). Finally, theoretical directions for the design of novel self-assembled (supra) colloidal systems are discussed.

## 1. Introduction

The physiological environment consists of “biological particles” such as proteins, organelles, viruses, cells, and a myriad of structures that move or are capable of self-assembling into tissues and organs [1]. These incredibly complex biological particles possess many very interesting properties such as surface chemistry, transport phenomena, catalytic activities, etc. [2]. A large majority of these “biological” particles result from the self-assembly of molecules into particles via a hierarchical sequential bottom-up construction in a given environment and conditions [3]. These particles are capable of self-assembling again into increasingly complex structures. For example, proteins have primary structures (defined as the sequence of amino acids linked together to form the polypeptide chain), secondary structures (defined as the local structures stabilized by H-bonds between certain amino acids of the polypeptide backbone and leading to α-helixes, β-sheets, and turns), and tertiary structures (defined as the overall 3D shape of a single protein molecule stabilized by nonlocal interactions such as hydrophobic forces, van der Waals interactions, salt bridges, and/or H-bonds) [4,5,6]. In addition to these three distinct aspects of protein structures, some of them have a quaternary structure, that is, the self-assembly of several polypeptide chains (subunits) to form a complex [7]. It is noteworthy that these four structures are relevant for proteins isolated under dilute conditions, but a quinary structure arises in vivo where protein surfaces are shaped by evolutionary adaptation to physiological conditions [8]. Most proteins self-assemble naturally and unassisted to form their native 3D ordered structures through non-covalent interactions. Generally speaking, self-assembly refers to the association of organic, inorganic, or hybrid entities, e.g., molecules, macromolecules, or nano-objects, into organized structures with nanometer to micrometer dimensions [9]. More specifically, in supramolecular chemistry, it refers to the process by which molecules spontaneously organize among themselves to form multimolecular structures whose stability relies on weak non-covalent interactions [10]. Self-assembly processes are essential in living organisms (e.g., the basic functions of the proteins are controlled by the tertiary structure) [11].

On the other hand, since van der Waals’ postulate on intermolecular forces in 1873, Fischer’s suggestion on enzyme–substrate interactions in 1894, and the description of the H-bond by Latimer and Rodebush in 1920, supramolecular chemistry has developed. With the elucidation of the structure of DNA and other fascinating biological entities, chemists began to emphasize the importance of non-covalent interactions. For example, the work of Pedersen, Cram, and Lehn (Nobel Prize in Chemistry in 1987) on crown ethers and cryptands allowed the design and synthesis of new highly selective molecular receptors. Following this work, research in the field has taken off rapidly with the work of Feringa, Stoddart, and Sauvage who received the Nobel Prize in Chemistry in 2016 for their work on molecular machines. In parallel, molecular self-assembly processes have found many applications, particularly in the development of new materials. Indeed, large structures are easily accessible using bottom-up synthesis because they are composed of small molecules requiring fewer synthesis steps. Currently, many smart materials are based on a bottom-up approach by molecular recognition. For example, in 1991, Simard et al. first introduced the concept of molecular tectonics, which offers a route for the bottom-up fabrication of predictable crystalline molecular networks from small molecules (tectons) through various interactions (van der Waals forces, electrostatic attraction, π-stacking, H-bonding, and coordination bonding) between the nearest neighbors [12]. The field of crystal engineering was born.

**Figure 1:**
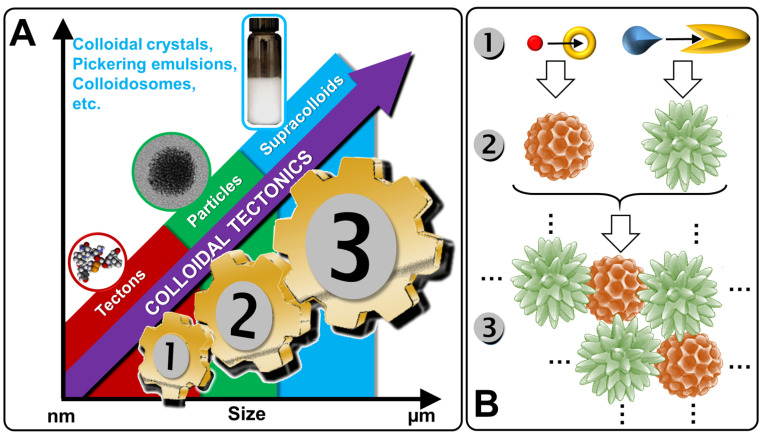
From tectons (molecules) to supracolloidal systems (**A**) and schematic illustration of the self-assembled process (**B**).

From a general perspective, there are two major types of self-assembled entities in living organisms: lipid assembly into dynamic, flexible, and fluid structures and protein assembly into rigid crystalline arrangements. The former is primarily driven by hydrophobic interaction while the latter is driven by a combination of hydrophobic, H-bonding, and electrostatic interactions. Lipid assembly is successfully mimicked by synthetic amphiphilic small molecules and polymers to form micellar, vesicular, and lamellar structures. In contrast, protein assembly that produces lamellae, tubules, and polyhedrons is largely unmatched by synthetic or nonpeptide molecules. The imbalance in lipid and protein mimicry research can be resolved using the colloidal tectonics approach. Indeed, since biological systems are a source of inspiration for chemists, the artificial construction by the self-assembly processes of molecules (including polymers), producing new abiotic particles with colloidal properties, refers to the concept of colloidal tectonics. This name was chosen by analogy with the pioneering work of Simard et al., which opened the field of crystal engineering [12]. In 1995, the same group proposed to define molecular tectonics as “*the art and science of supramolecular construction using tectonic subunits*” [13]. This concept has been extended to liquid crystals [14]. In 2018, I proposed the name colloidal tectonics, the self-assembly of complementary molecules leading to a variety of particles or rigid objects with colloidal properties (i.e., producing dispersions) [15]. This concept also includes interactions between objects giving rise to supracolloidal assemblies (e.g., Pickering emulsions, colloidosomes, etc.) since they emerge from the packing of particles under appropriate conditions [15]. However, all these tectons (molecules or particles) have complementary attractive sites allowing a bottom-up construction of large colloidal systems (down to the microscale) through non-covalent interactions driven by internal (e.g., hydrophilic/hydrophobic ratio, charge, H-bond donor and/or acceptors sites, and molecular conformation) and external factors (i.e., environmental conditions surrounding the nanostructure such as pH, concentration, temperature, and solvent) [15]. The assembling process is based on a recognition operating at the level of complementary tectons (molecules or particles) providing an infinite variety of (supra) colloidal systems [15]. In the strict sense of the term self-assembly, the resulting systems are intrinsically predictable, versatile, switchable, and can even be dissociated in their constitutive elements. Thus, over the last decade, new smart colloidal tectonics systems have been developed [15]. This approach is operational and versatile since it relies on self-assembly processes resulting from recognition events between programmed building blocks (molecules and/or particles), known as tectonic subunits or simply tectons. These events enable the design and construction, under mild conditions, of a variety of complex colloidal systems that are highly compatible with green chemistry. Indeed, all these systems are energy efficient, minimizing synthesis steps and harmful organic solvents [15]. As these abiotic particles can have the same properties as their biotic cousins (i.e., surface chemistry, transport phenomena, catalytic activities, etc.), all applications requiring the colloidal engineering of nanometric and micrometric systems can be envisaged, such as catalysis, cosmetics, drug delivery, vaccination, etc. [16]. All these systems, in which a bottom-up construction of large colloidal systems is allowed from tectons by simple molecular recognition, result in nanostructured systems and constitute a new and versatile field with promising green assets in the face of environmental problems (Figure 1) [15].

In this review, I focus on the fundamentals of colloidal tectonics by illustrating some self-assembled biological colloidal systems. The design of smart and switchable abiotic (supra) colloidal systems and their applications in various fields are also discussed (with a particular focus on Pickering emulsions for catalysis or drug delivery). Finally, theoretical directions for the design of novel self-assembled (supra) colloidal systems are discussed. I hope that this review can offer new insights and provide theoretical guidance in the design of novel self-assembled (supra) colloidal systems.

## 2. Colloidal Tectonics: A Bioinspired Concept

As is often the case, nature already uses the concept of colloidal tectonics to obtain supramolecular colloidal structures. As these ingenious biological systems can serve as inspiration for chemists, a few biotic self-assembled colloidal systems are presented in this review. It should be noted, however, that, for the sake of clarity, only a few examples are reported in this section. Therefore, the examples cited are not intended to constitute an exhaustive list.

### 2.1. Milk Casein Particles

Milk is the lactic secretion of mammals and contains valuable nutrients and immune components for the optimal growth of newborns [17]. Although the composition of milk varies depending on the stage of lactation and the growth rate of the newborn, the essential components include water, lipids (fatty acids, phospholipids, and cholesterol), vitamins, carbohydrates (lactose), minerals (calcium), and proteins (phosphoproteins and enzymes), thus contributing to the energy needs of newborn growth [18]. The most important proteins found in mammalian milk are caseins (phosphoproteins), which represent approximately 20 and 80% of the proteins in human and cow milk, respectively. Regardless of the type of mammal, there are four types of caseins (α_S1_, α_S2_, β, and κ) [19]. The most important amino acids present in casein isolates are, in order of importance, glutamic acid, proline, leucine, lysine, valine, and aspartic acid [20]. Caseins are non-crystallizable proteins, as the high number of proline acts as a structural disruptor preventing local compaction (i.e., the formation of secondary structural motifs common to proteins) and inhibiting the formation of ordered 3D structures (i.e., tertiary structure) [21]. The presence of hydrophobic (proline, leucine, and valine) and hydrophilic (lysine, glutamic acid, and aspartic acid) amino acids gives caseins an amphiphilic nature but the absence of a tertiary structure makes them poorly soluble in water due to the inevitable exposure to many hydrophobic residues. Therefore, milk is a physically stable aqueous suspension of caseins due to their strong self-association to form supramolecular particles called casein micelles [22]. Classically, the term “micelle” describes a supramolecular assembly of amphiphilic molecules (surfactants) leading to a colloidal suspension in which the hydrophilic “heads” of the surfactants reside on the surface in contact with the surrounding water, sequestering the hydrophobic “tails” inside the micellar core [23]. However, the core of surfactant micelles is not hydrated, unlike casein micelles (see below) [24]. The term “casein micelle” is therefore erroneous because the only similarity with surfactant micelles is that the hydrophilic parts are on the surface. Indeed, casein micelles should rather be considered as colloidal spherical particles of 20 to 600 nm in diameter (the average diameter is about 120 nm) composed of several thousand associated caseins [25]. It should be noted that κ-caseins are more hydrophilic than the other three and are therefore predominant on the surface of casein micelles while the more hydrophobic ones are found inside [26].

**Figure 2:**
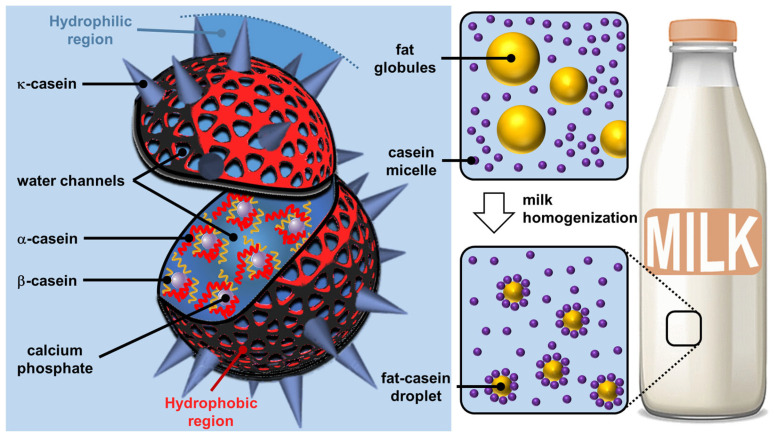
Structure of casein micelles (**left**) and effect of homogenization on fat and casein fractions in milk (**right**).

Although caseins have been known for nearly 70 years as unfolded proteins, the implications for the structure of casein micelles are still debated. In 1965, Waugh and Noble proposed a core–shell model consisting of spherical hydrophobic casein particles surrounded by κ-casein [27,28]. In 1966, Payens hypothesized that κ-casein formed the shell of the casein particles and that the compactly folded α_S1_- and α_S2_- caseins were attached to the free β-caseins (core), with calcium ions interacting with the phosphate or carboxylic acid groups of the proteins [29]. From the model of Slattery and Evard (1973), which excluded the function of colloidal calcium phosphate [30], Schmidt and later Walstra proposed, in 1980 and 1990, that the casein micelle was built by 10 to 100 submicelles (4 to 10 nm in diameter) [31,32]. Each submicelle is composed of a hydrophobic core containing mainly α_S1_-, α_S2_-, and β-casein, whereas the shell contains hydrophilic regions composed of the phosphate groups of α_S1_-, α_S2_-, β-caseins, and a variable proportion of κ-casein. The submicelles low in κ-casein are located on the surface to ensure solubility in whey while the others are at the core of the casein micelle. Since casein proteins are able to interact with colloidal calcium phosphate, Schmidt assumed that colloidal calcium phosphate filled the space between the submicelles. In 1999, this model was refined by Walstra, who proposed that colloidal calcium phosphate was located within the submicelles and not only between them [33]. In 2012, Dalgleish and Corredig hypothesized that the casein micelle is not based on submicelles but rather on a matrix structure with colloidal calcium phosphate nanoclusters surrounded by calcium-sensitive caseins (α_S1_-, α_S2_-, β-caseins) while the κ-caseins protect the casein micelle surface to ensure solubility in the aqueous environment and inhibit the growth of casein micelles by steric repulsions [34]. Interestingly, there are several other models that account for the spatial conformation of casein in micelles [35]. One of them proposes a double link between caseins for gelation to occur [36]. Fortunately, all models consider casein micelles as colloidal particles formed by casein aggregates wrapped up in hydrosoluble κ-casein molecules (Figure 2).

Despite the debate over its exact structure, casein micelle is built around strong interactions with calcium phosphate nanoclusters, while weaker, multivalent casein–casein interactions make casein micelles highly dynamic structures that complicate their modeling in the complex milk environment. Regardless of their exact structure, casein micelles are supramolecular colloidal assemblies with a lattice structure in which casein polymers and casein–calcium phosphate aggregates act together to maintain structural integrity through hydrophobic and electrostatic forces (i.e., calcium phosphate bridges), preventing spatial changes and the dissociation of the assembled structure unless the chemical environment is altered [37]. Unlike surfactant micelles, the core of casein particles remains highly hydrated: they are open sponge-like particles that are generally resistant to spatial changes due to their interlocked structure and the presence of multiple interactions [38]. Nevertheless, the structural arrangement within the casein particle can be altered by environmental factors that modify hydrophobic interactions and calcium phosphate solubility (e.g., pH, temperature) [39]. The spontaneous bottom-up edification of casein micelles using the interactions between casein proteins and calcium phosphate can be seen as tectonic subunits providing (supra) colloidal structures. The formation is driven by the interactions between the hydrophobic regions of the caseins and the phosphocalcic bridges. The self-assembly of casein micelle can be interpreted by calcium phosphate phase separation. In 2024, Antuma et al. supposed that the caseins would interact with the seed of calcium phosphate formed by pre-nucleation through their phosphoserine residues and subsequently self-assemble to form casein micelles, inhibiting the crystallization of calcium phosphate (i.e., the maturation into crystalline phases) [40]. Casein micelles give rise to a porous structure in which various other molecules or solvents can be inserted. These particles lead to Pickering-like emulsions (i.e., emulsions stabilized by particles absorbed at the water/oil interface) after milk homogenization (i.e., a process used to mix and disperse milk fat globules to avoid phase separation and achieve a homogeneous texture, hence the name of the process) when the casein micelles replace the milk fat globule membranes, which are damaged during homogenization (Figure 2) [41].

### 2.2. Plant Latex Particles

Another archetypal example of natural colloidal particles is found in plant latexes (i.e., wet rubber). Natural latex is a stable milky white, yellow, orange, brown, or even colorless colloidal dispersion of various particles (organic and inorganic) consisting of tiny droplets of organic matter dispersed in water [42]. Latex is present in at least 20,000 plants (with a high prevalence in flowering plants that form the angiosperm clade) and in several fungi such as *Lactarius deliciosus* [43]. The major botanical families producing latex in most of their species are *Euphorbiaceae* (which includes *Hevea*), *Moraceae* (especially *Ficus*), *Apocynaceae*, *Campanulaceae*, *Papaveraceae*, *Sapotaceae*, *Asclepiadaceae*, *Convolvulaceae*, *Asteraceae*, etc. [44] Latex is produced by extremely elongated secretory cells called laticifers constituting the laticiferous system [45]. Latexes arise from vacuoles occupying a very large part of the laticiferous cells. It should not be confused with plant sap because their functions are clearly different [45]. Indeed, after tissue injury, latex, which is the plant’s first line of defense, coagulates upon exposure to air, serving mainly as a natural defense mechanism against herbivorous insects and other pathogens while sap ensures the distribution of water, mineral salts, or sugars [43]. It circulates in a distinct network of vessels. Indeed, latex forms a barrier against pathogen invasion due to its sticky and coagulating properties, limiting pathogen movements. Despite the variability of latex components, some chemical species can be distinguished, such as rubber, terpenoids, alkaloids, cardenolides, carotenoids, carbohydrates, phenolics, furanocoumarins, various metal ions, and proteins [46]. Some of them are toxic and/or dissuasive components depending on the plant species concerned.

**Figure 3:**
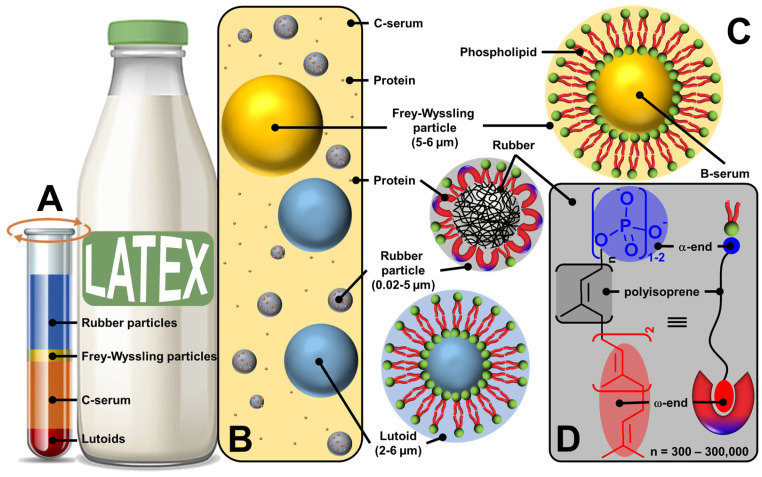
Ultracentrifuged fresh natural rubber latex fractions (**A**), schematic representation of liquid fresh rubber latex (**B**), structure of rubber and non-rubber particles (lutoid and Frey–Wyssling particles) (**C**), commonly accepted polyisoprene molecular structure (**D**) indicating the self-binding between the α-end and phospholipids and the ω-end and proteins through electrostatic and hydrophobic interactions (bottom right).

In the following discussion, we focus on rubber latex, harvested from the rubber tree (*Hevea brasiliensis*) that grows in tropical regions, which is called fresh latex. Fresh latex is a stable colloidal dispersion of a polymeric substance (rubber) in a serum, which consists of phospholipids, proteins, inorganic salts, etc. In natural rubber, the polymeric substance is cis-1,4-polyisoprene. Fresh natural rubber latex is a whitish color fluid containing 30–33% natural rubber, 1–1.5% protein, 60–65% water, and some resinous substances [47,48]. After ultracentrifugation, fresh latex can be separated into four fractions (Figure 3) [49,50].

The upper layer (35%) is composed of 86% rubber particles dispersed in an aqueous medium, 3% lipids (sterol, sterol esters, waxes, and phospholipids), 1% proteins (α-globulin and hevien), and 0.05% metal ions such as Mg, K, and Cu. The shape of the rubber particles varies from spherical to oval depending on the age of the tree, and their size varies between 0.02 and 5 µm [42]. The second yellow or orange fraction (5%) contains Frey–Wyssling particles, which are composed of a lipid bilayer sequestering carotenoid pigments (giving them a yellow-orange color), oxidative enzymes, and proteins [42]. The third fraction (50%) corresponds to the centrifuged serum (C-serum) constituting the cytoplasm of laticifers and contains about 60% of all fresh latex proteins [42]. C-serum contains carbohydrates (methylinositol), water-soluble proteins (hevien), free nitrogenous bases, organic acids, inorganic anions (PO_4_^3−^, CO_3_^2−^), and metal ions (K, Mg, Fe, Na, and Cu). It is noteworthy that C-serum contains proteins that contribute to the colloidal stability of latex and rubber particles (see below). The lower fraction (10%) contains vacuole-like organelles called lutoids. Structurally, lutoid particles are composed of a lipid bilayer sequestering a fluid known as B-serum, which contains hydrolytic enzymes and proteins, and latex agglutination factors involved in the agglutination processes of latex particles [42]. Thus, fresh latex of *Hevea brasiliensis* contains three types of particles: rubber particles, present in the upper fraction; Frey–Wyssling particles, present mainly in the small second yellow or orange fraction; and Homans and van Gils lutoid particles in the lower fraction (Figure 3C). Since these rubber and non-rubber particles (lutoid and Frey–Wyssling particles) are dispersed in a continuous aqueous phase (i.e., serum C), fresh latex is a stable colloidal system [51].

**Figure 4:**
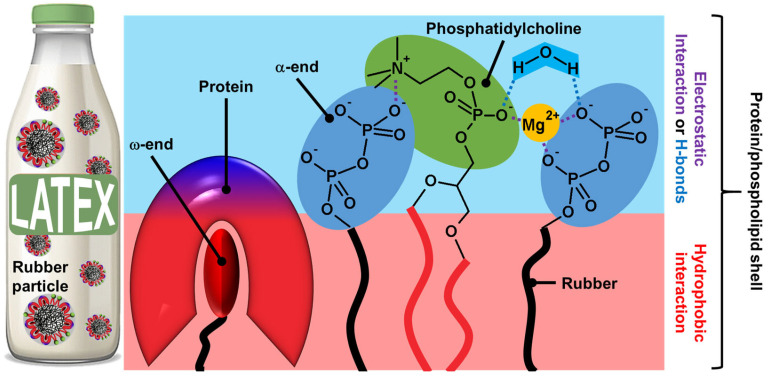
Presumed structure of rubber particles shell and non-covalent interactions between phosphatidylcholines and proteins (electrostatic and hydrophobic interactions).

On the other hand, the microstructure of polyisoprene molecules is known to have two terminal chain ends: α- and ω-ends (Figure 3D) [52,53]. Indeed, chain elongation in natural rubber latex biosynthesis caps one end of the rubber molecule with a mono- or diphosphate group (α-end), while the other end is capped with two *trans*-isoprene units (ω-end). The ω-end is linked to proteins resulting from the biosynthetic process probably via hydrophobic interactions. Indeed, both proteins (i.e., REF and SRPP) present a group of hydrophobic amino acids creating pockets and/or nonpolar surfaces, which allow hydrophobic interactions with the ω-end of polyisoprene molecules [54,55]. The association between the α-end and phospholipids occurs through direct electrostatic interactions or is mediated by Mg^2+^ ions or H-bonds with water molecules (see Figure 4).

Indeed, the phosphate groups are bonded to water via H-bonds within the surface, leading to a cooperative network [56]. The polyisoprene chain with the protein–phospholipid linkage can be moved and connected together as a network according to the reptation theory by de Gennes, leading to core–shell particles [57]. The phospholipid-protein layers are held on the surface of rubber particles by specific interactions. Indeed, proteins and phospholipids interact in the shell through hydrophobic contacts due to the clustering of hydrophobic amino acids in REF and SRPP (see above). It should be noted that electrostatic interactions also stabilize the shell. If there is no clear clustering of positive and negative residues in REF and SRPP, folding may occur in the presence of phospholipids to create charged surfaces and/or pockets that favor contacts with the phospholipid headgroup. In addition to the formation of direct ionic bonds between the negative charges of phospholipids, the polar head group of phospholipids participates in H-bonds with the polar solvent (i.e., water molecules) that restrict the mobility of the phosphate group, also stabilizing the mixed shell [56,58]. All the colloidal particles of fresh latexes allow the formation of amphiphilic regions that can be used as emulsifiers, leading to Pickering-type emulsions. Kumar and Basavaraj reported that plant latexes offer a sustainable route to reduce oil/water interfacial tension due to the spontaneous adsorption of surface-active species present in the latexes [59]. The surface-active species include molecular emulsifiers and particles. Indeed, under a cross-polarized microscope, the droplet surface is birefringent due to the interfacial adsorption of solid particles present in the latex. This mixed particle/surfactant emulsifier system of biological origin avoids the use of molecular surfactants involving complex synthesis routes, high production costs, and the generation of chemical waste.

### 2.3. Viral Particles

**Figure 5:**
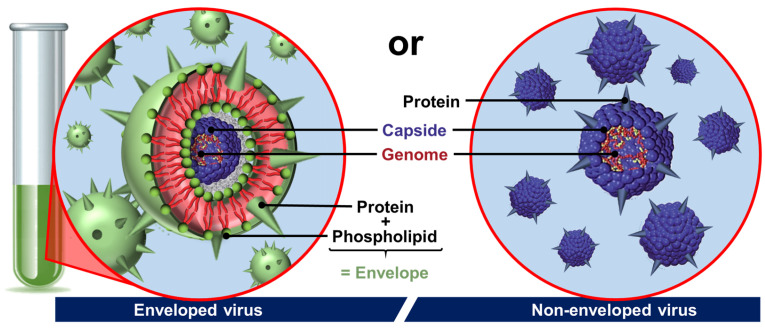
Schematic drawing of two basic types of viruses. In non-enveloped viruses, the genome is condensed in a capsid (coat protein), whereas enveloped viruses have a capsid or nucleocapsid wrapped in a phospholipid bilayer with protein (spike).

The last archetypal example of natural colloidal particles is found in viruses. Indeed, viruses are infectious agents, generally nanometric and stable in air or water, which use host cells to multiply. After contact and internalization in a target cell, the virus disassembles to transfer its genome, which is then translated into new viral components without their knowledge. Then, the viral components spontaneously self-assemble into new viruses that leave the cell to infect others, and the viral cycle begins again. The assembly and disassembly mechanisms are therefore closely linked to the proliferation of viruses and probably represent the most advanced case of biological supramolecular structures [60]. Viruses are composed of (i) the genome consisting of a nucleic acid chain (DNA or RNA); (ii) the capsid which surrounds and protects the genome from the external environment by a semi-rigid protein shell; and, sometimes, (iii) the envelope that covers the capsid and contains lipids and proteins essential for their binding to target cells (Figure 5) [61].

**Figure 6:**
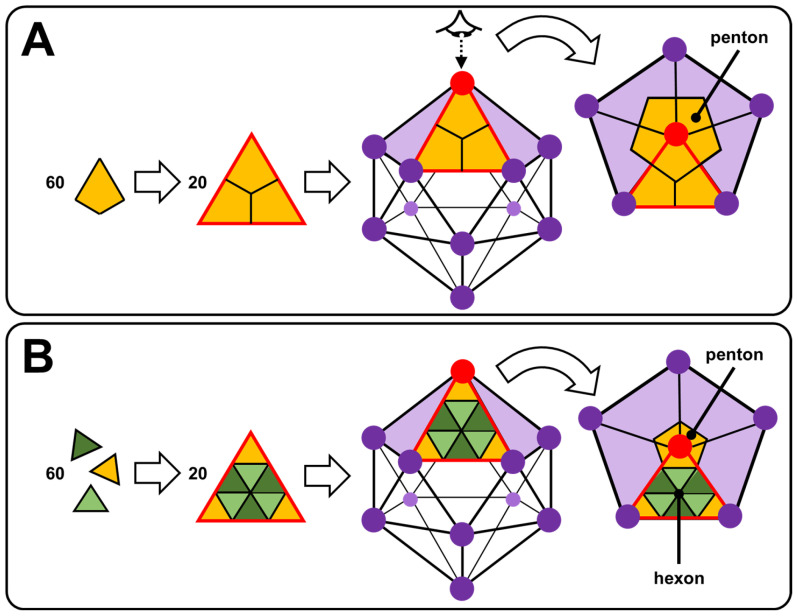
Self-assembly of 60 T asymmetric units (proteins) giving 20 triangular facets, leading to icosahedral nucleocapsid with T = 1 (**A**) and T = 3 (**B**).

The most fascinating part of viruses is probably the capsid. Many capsids are icosahedral or nearly spherical with icosahedral symmetry as anticipated by Watson and Crick in 1956 and, more elaborately, by Caspar and Klug in 1962 [62,63]. The exterior of an icosahedral capsid is composed of repeating protein subunits (capsomeres). The regular icosahedron, characterized by 5:3:2 rotational symmetry, is the best way to form a closed shell from identical subunits: the proteins constituting the capsid are arranged on the faces, edges, or vertices of an icosahedron [64]. In more detail, the regular icosahedron is composed of 20 triangular faces and 12 vertices. Viruses with icosahedral symmetry contain 60 T proteins (where T is the number of distinct protein configurations), forming 12-vertex pentons (pentameric capsomers) and 10(T − 1) hexons (hexameric capsomers). For T = 1, the capsid contains only 12 pentagonal motifs (Figure 6A). In Figure 6B, the proteins have an identical chemical nature but with three different conformations (T = 3) identified by three colors leading to a capsid with 12 pentons centered on the vertices of the icosahedron (yellow proteins) and 20 hexagonal motifs (alternating dark and light green proteins). Thus, for larger viruses, the additional capsomers are arranged in a regular hexagonal lattice on the faces of the icosahedra. For instance, the cauliflower mosaic virus has an icosahedral capsid built from 420 proteins (12 pentons and 60 hexons): it is a T = 7 virus that obeys the rules of Caspar and Klug [65]. Icosahedral symmetry can be understood by simple geometric arguments: a structure consisting only of hexagons would have a maximum surface density, but it could not be closed because it would be flat. To introduce the curvature required for a closed structure, one must have exactly twelve pentagons, with the rest of the structure being composed of hexagons. If these twelve pentagons are distributed equidistantly, the structure exhibits icosahedral symmetry. However, when the twelve pentagons imposed by the topology are distributed non-uniformly, new geometric shapes are observed, and icosahedral symmetry is lost [65]. This is the case, for example, of the human immunodeficiency virus type 1 (HIV-1) or the tobacco mosaic virus (TMV), whose capsids are, respectively, conical or helical in shape [66,67].

Although our knowledge of genome packaging in a capsid is still embryonic, most capsids are formed by a self-assembly process that can be reproduced in vitro by mixing the genome with the subunits in an aqueous medium. These are often protein dimers because they are more stable than the proteins alone. This process is favorable (i.e., spontaneous) because the energy gain linked to the association of the subunits is greater than the entropy loss that is also associated with it. There is, however, a threshold concentration of subunits for self-assembly to be initiated. The energy gain is associated with non-covalent interactions between subunits (van der Waals and/or hydrophobic forces). After a transient regime, the association between the proteins eventually reaches a state of chemical equilibrium governed by a mass action law [68]. The genome, however, helps guide assembly. Protein–genome interactions are primarily electrostatic in origin because proteins often contain positively charged chemical groups, whereas the genome invariably carries negative charges. Indeed, there is a linear relationship between the total electrostatic charge of the genome and that of the protein subunits [69]. It is noteworthy that empty capsids (i.e., without genome) can also be formed under appropriate ionic conditions.

The self-assembly of an empty capsid is often interpreted in terms of nucleation/growth [70]. It should be noted that the term nucleation refers to a process by which atoms or molecules aggregate into a small unstable seed, from which a larger assembly will grow. Originally observed for crystal formation, nucleation appears to be a generic pathway, including many molecular assemblies [71]. The subunits begin by forming a seed under the action of a local concentration fluctuation; this is the nucleation phase. Other subunits that remain free then bind sequentially to this seed to make it grow rapidly until the formation of a complete capsid. For example, the seed is a trimer of subunits for the capsid of the hepatitis B virus [72]. Unfortunately, the seed varies according to the virus considered and remains difficult to identify due to its transient nature. The lower the free energy of association, the greater the number of subunits constituting the seed will increase. In addition, assembly and disassembly exhibit significant hysteresis that allows the assembled capsids to remain metastable even at high dilution, which is a crucial property for their survival outside the intracellular environment. In the presence of a genome, two scenarios are possible, depending on the interaction energies between the components: (i) the capsid assembles according to the nucleation/growth mechanism (see above) while simultaneously packaging the genome, or (ii) the subunits rapidly bind to the genome and then cooperatively reorganize into a capsid [73].

Some virus particles allow the formation of emulsions. For instance, the icosahedral capsids of cowpea mosaic virus (CPMV) and turnip yellow mosaic virus (TYMV) have the ability to stabilize Pickering emulsions [74,75]. However, viral envelope proteins can also be used to obtain long-term stable Pickering emulsions. For example, the amphiphilic envelope proteins of TMV can self-assemble at Pickering emulsion interfaces [76]. In all these cases, robust capsules can be fabricated by cross-linking between the coat proteins. By taking advantage of the amphiphilicity of coat proteins separately or directly on the capsid, the construction of robust Pickering membranes can have various applications, including drug delivery or virus recognition.

### 2.4. Nature and Colloidal Tectonics

All living organisms exhibit self-assembled entities. The three examples, presented in this section, show a core–shell structure with at least one layer of proteins (i.e., building blocks or tectons) surrounding the core. For casein micelles, the core is composed of hydrophobic proteins (α- and β-caseins) while the coat is composed of the more hydrophilic κ-caseins. For rubber particles, a mixed layer of proteins and phospholipids surrounds the hydrophobic polyisoprene core. For non-enveloped viruses, the nucleic acid is surrounded by capsid proteins. In all three systems, the main driving force of particle formation is hydrophobic forces supported by H-bonds and electrostatic interactions (relatively strong interactions). It should be noted that other biomolecules form self-assembled entities. For instance, lipids can self-assemble into membranes through primarily hydrophobic interactions, a relatively weak and non-directional interaction [77]. However, these systems are typically soft, fluid, and less-ordered (despite the coexistence of soft fluid and rigid gel domains in plasma membrane), whereas proteins self-assemble into rigid crystalline structures [78]. In general, chemists can easily reproduce lipid assemblies using synthetic amphiphilic small molecules and/or polymers to form micelles, vesicles, and/or lamellar structures [79]. In contrast, protein assemblies that produce assemblies with structural rigidity and crystallinity (e.g., lamellae, tubules, and/or polyhedrons) are more difficult to mimic by synthetic molecules [80]. Colloidal tectonics deals with the formation of artificial rigid and crystalline colloidal structures, similar to protein assemblies, which is a fundamental challenge in materials science [15].

In the three previous examples, the self-assembly of tectons into particles can be interpreted in terms of nucleation/growth. Indeed, some tectons start by forming a seed under the action of a local concentration fluctuation (nucleation) and then other tectons that remain free sequentially bind to this seed to make it grow rapidly until the formation of a complete particle. This process results in colloidal particles constituting hydrophilic and hydrophobic regions in which solvent(s) can be accommodated or at least interacted. The spontaneous formation of colloidal structures from tectonic subunits depends on the total interaction energy (i.e., the sum of attractive and repulsive interactions) [15]. The structure of the tectons influences the physicochemical properties of the particles and thus the possibility of obtaining supracolloidal structures such as dispersion, Pickering emulsions, colloidal crystals, etc. [15]. There is therefore a continuous transition between the properties of the tectons and those of (supra) colloidal systems [15]. In the first publication on the concept of colloidal tectonics, the following guidelines were proposed to obtain a bottom-up colloidal construction: two or more complementary tectons (i.e., with suitable binding sites to form supramolecular seeds essential for the growth of the colloidal particles) of opposite polarities, at least one of which has a rigid structure [15]. However, more recent papers have supplemented and refined these guidelines (see below). Indeed, the new formulation is as follows: colloidal systems can be generated using either a self-complementary tecton (single-component system) or two or more complementary tectons (multicomponent system). In addition to intermolecular interactions, structural flexibility/rigidity and fluidity/crystallinity are also essential for the spontaneous bottom-up formation of colloidal structures.

**Figure 7:**
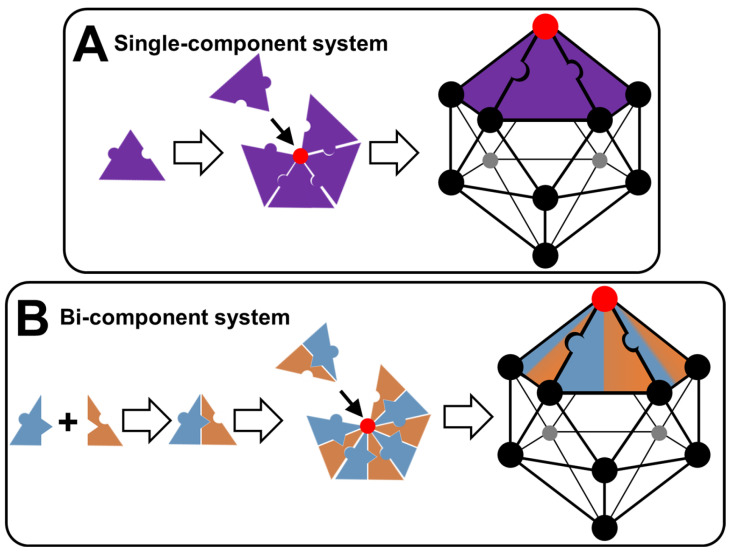
Schematic representation of colloidal arrangements in the form of regular icosahedrons (capsid-like structures) made with a single- or bi-component system (**A**) or (**B**).

Let us focus here on the single-component system, supposing that a capsid-like structure forms. The tecton presented in Figure 7A has both polar and nonpolar binding sites. Due to this self-complementarity, tectons lead, under appropriate conditions (temperature, concentration, solvent, etc.), to the formation of pentamers (i.e., pentagonal pyramid). The mutual interconnection of pentamers is achieved by complementary interactions leading to a regular icosahedral envelope. The resulting polyhedron has 20 equilateral triangles as its faces, 30 edges, and 12 vertices. Therefore, two levels may be used to describe the growth of icosahedron colloidal arrangements: the formation of pentamers and the self-association of pentamers. The second possibility (Figure 7B) concerns the formation of icosahedrons using a two-component system based on two tectons of opposite polarity. However, the two tectons can be paired due to complementary attractive interactions forming discrete supramolecular structures. These structures can form pentamers by mutual bridging. For reasons of compaction, pentamers are grouped in regular icosahedrons (see above). In this case, three levels may be used to describe this system: the establishment of discrete supramolecular structures, the formation of pentamers, and the lateral association of pentamers leading to regular hollow icosahedrons. From a general point of view, intermolecular interactions, structural flexibility/rigidity, and fluidity/crystallinity are key parameters, as well as the morphology of tectons. Indeed, tectons of identical chemical nature can adopt different conformations allowing them to obtain pentagonal patterns and hexagonal patterns, leading to icosahedral symmetries. The self-assembly process is favorable and therefore spontaneous because the energy gain of association of the subunits is greater than the entropy loss that is also associated with it. However, there is a threshold concentration of subunits for self-assembly to be initiated. The energy gain is associated with short-range attractive interactions between the subunits (hydrophobic and/or van der Waals forces). After a transient regime, the association between the subunits finally reaches a state of chemical equilibrium governed by a mass action law. This self-assembly is often interpreted in terms of nucleation/growth (see above).

Colloidal tectonics deals with the design and generation of colloidal systems based on tectons, which are active building units carrying recognition information and thus capable of recognizing each other. Colloidal arrangements are formed under self-assembly conditions, leading to reversible and adaptable systems with self-repair capacity. In other words, complementary tectons find their own way to the most stable situation under the given conditions (temperature, concentration, solvent, etc.). The main recognition events are solvophobic interactions, which may be complemented by a variety of other reversible attractive intermolecular interactions such as van der Waals, electrostatic forces, and H-bonds [15]. However, structural flexibility/rigidity and fluidity/crystallinity are also essential parameters (see above). However, unlike the concept of molecular tectonics [12,13], which deals with the design and preparation of infinite periodic molecular networks formed under self-assembly conditions between self-complementary or complementary tectons, the growth of colloidal tectonics assemblies is inhibited by various repulsions such as steric and electrostatic repulsions [15]. Therefore, a simple design rule for colloidal tectonics-based systems is the presence of (i) attractive interactions conferring rigidity and/or crystallinity, and (ii) repulsive forces to avoid the long-range order characteristic of a crystal. Since colloidal systems play a crucial role in nature and in many scientific and industrial applications, some recent examples of strategies to obtain such systems using colloidal tectonics are presented in the following section, together with their uses.

## 3. Design and Applications of Artificial Architectures Based on Colloidal Tectonics

In this section, it is noteworthy that artificial architectures based on colloidal tectonics are not presented from a historical perspective. Indeed, I have discarded this view to use a classification according to the number of components involved in the colloidal edification. Indeed, architectures can be generated using either a self-complementary tecton (single-component system) or two or more complementary tectons (multicomponent system). In each case, subdivisions are made according to the chemical nature of the tectons. It should be noted that, for the sake of clarity, only a few examples are reported in this section, with particular emphasis on Pickering emulsions for catalysis or drug delivery.

### 3.1. Single-Component Colloidal Systems

Colloidal tectonics can be used to generate colloids by self-assembly processes involving a single self-complementary tecton. Such a single-component system is the most attractive situation in terms of atoms and synthetic economy. Since the most important requirement is to have solvophobic (i.e., hydrophobic and organophobic) sections, solvophobic block copolymers can be good candidates. Indeed, the low solubility of one of the blocks provides a sufficient driving force for their self-assembly in a given solvent. In such aggregates, the solvophobic blocks associate into dense central domains surrounded by solvated coronas formed by solvophilic blocks. This driving force is counterbalanced by repulsive interactions between solvophilic blocks that ensure the formation of stable self-assemblies at the nanoscale. For example, in aqueous solutions, the assembly is clearly driven by the hydrophobic attraction between the associated blocks and counterbalanced by electrostatic or steric repulsions between ionic or neutral organophobic blocks. However, to avoid the formation of dynamic polymeric micelles similar to classical molecular surfactants, one has to keep in mind that the polymer must have structural flexibility/rigidity and fluidity/crystallinity in addition to complementary intramolecular interactions (see above). To manage flexibility/rigidity and fluidity/crystallinity, additional interactions have to be used, such as H-bonds, aromatic stacking, van der Waals interactions, etc. In practice, homopolymers and surfactant-based systems can also be used. In this section, some typical examples are given to illustrate the effectiveness of these approaches.

#### 3.1.1. Homopolymers

**Figure 8:**
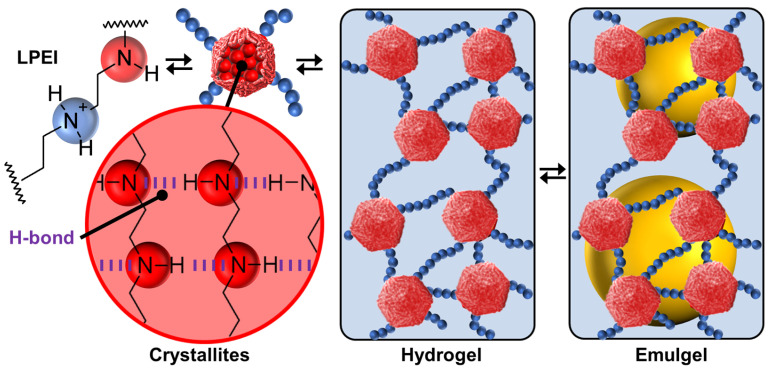
Structure of linear polyethyleneimine, LPEI, with protonated and unprotonated amine groups, leading to crystallites formed by H-bonds between unprotonated amine groups, thermo-reversible hydrogels, and gelled emulsions (emulgels).

In 2022, Douyère and his collaborators reported a system based on linear polyethyleneimine (LPEI) as a unique self-complementary tecton to design self-assembled gelled emulsions (i.e., emulgels) [81]. The polymer has a high molecular weight to obtain an appropriate rigidity/flexibility balance. The hydrophilic-lipophilic balance is obtained by the protonation/deprotonation of the amine groups. Indeed, depending on the LPEI protonation, the polymer is in a free form at pH < 2.3 or fully aggregated at pH > 10, leading, respectively, to a solution or an LPEI dispersion. Between these two behaviors, LPEI acts as a buffering agent by the continuous protonation/deprotonation of the amine groups. Thus, in an aqueous solution, LPEI molecules give a switchable gel depending on the pH and temperature. The formation of thermo-reversible hydrogels is due to adequate and spontaneous distributions of “hydrophobic” and “hydrophilic” regions on the LPEI chains (Figure 8). Indeed, the “hydrophilic” regions are protonated amine groups while the “hydrophobic” regions are neutral -CH_2_CH_2_NH- units. The gel phase is obtained by a pseudo-crystallization of “hydrophobic” regions, under the action of hydrophobic forces and H-bonds, while the protonated regions remain hydrated (Figure 8). In an aqueous solution, competition between hydrated and associated regions results in the formation of a 3D network where the crystallized hydrophobic domains act as knots (Figure 8). An interesting comparison can be made with poly(4-vinylpyridine), P4VP. Indeed, linear P4VP, even partially protonated, is not able to provide crystallites or other self-assembled colloidal entities capable of stabilizing Pickering-like emulsions [82]. In contrast, acidic colloidal particles of P4VP (39 mmolH+/g) cross-linked with 2% divinylbenzene (Reillex^®^ 402 ion-exchange resin) are able to give heptane-in-water Pickering emulsions with long-term stability [83]. Recalling these observations, a simple design rule for self-assembly can be put forward: the presence of strong attraction is mandatory (H-bonds for LPEI molecules and cross-linking, involving the formation of covalent bonding between adjacent polymer backbone for P4VP particles). However, it is noteworthy that acidic P4VP particles containing 25% divinylbenzene (Reillex^®^ 425) do not lead to hydrogels or Pickering-type emulsions. A higher cross-link density is the result of a greater number of bonds per polymer chain length, which results in greater rigidity and hardness, proving the importance of structural flexibility/rigidity and fluidity/crystallinity compared to morphology [82]. Indeed, the 2% cross-linked protonated P4VP particles have a spongy structure, leading to solvent penetration capable of interacting with the hydrophobic regions of P4VP. Therefore, the cross-linked systems are less adaptable than LPEI-based systems that take advantage of reversible H-bonds to adapt to the external environment.

As LPEI provides a robust tecton with solvophilic and solvophobic regions, leading to structural flexibility/rigidity and fluidity/crystallinity, the spontaneous bottom-up self-assembly provides a good platform to obtain Pickering-like emulgels [81]. Since LPEI is not soluble in either conventional organic solvents (heptane, toluene, isopropyl myristate, and liquid paraffin) or vegetable oils (corn, sunflower, olive, and castor oil), the authors obtained long-term stable emulgels after the homogenization of biphasic oil/water systems (1:1 *w*/*w*) in the presence of LPEI [81]. Indeed, the stabilizing mechanism of the obtained systems is unusual: (i) crystallites are homogenously distributed on the surface of the droplets, leading to Pickering-like stabilization, and (ii) crystallites act as knots, contributing to the gelation of the aqueous continuous phase and the emulsion’s stability (Figure 8). Moreover, due to their self-assembled nature, these emulgels provide reversible systems with a high degree of control. Indeed, sol/emulgel transitions are obtained by temperature or pH changes because the acidic environment and/or high temperature alter the self-assembly of the “hydrophilic” and “hydrophobic” regions, leading to destabilization by modifying the gelation of the aqueous continuous phase and the Pickering stabilization (alternating stabilization and phase separation up to 10 consecutive runs) [81].

#### 3.1.2. Copolymers

**Figure 9:**
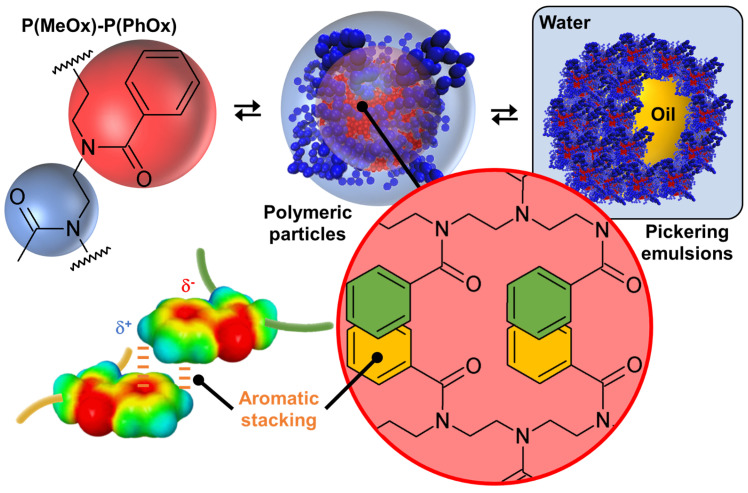
Structure of copoly(2-methyl/phenyl-2-oxazoline), P(MeOx)-P(PhOx), polymer particles formed by stacking interactions between phenyl side groups, and resulting Pickering emulsion.

Since it is possible to obtain tectons from homopolymers when the latter possess ionizable functions, the use of amphiphilic copolymers has been considered. For example, Bardoula and his collaborators reported the use of amphiphilic polymer particles based on copoly(2-methyl/phenyl-2-oxazoline)s, P(MeOx)-P(PhOx), where MeOx is the hyrophilic monomer and PhOx is the hydrophobic monomer, to obtain Pickering emulsions (Figure 9) [84]. Well-defined polymer particles are obtained by nanoprecipitation. The effects of chain length (degree of polymerization, DP = 50, 100, 150, or 200) and monomer distribution (block or gradient) on the properties of the polymer particles are also investigated. For a fixed MeOx/PhOx ratio of 25/75 (*w*/*w*), the intensity-weighted average hydrodynamic size of all particles measured by dynamic light scattering ranges from 69 ± 15 to 152 ± 10 nm for block copolymers and from 65 ± 3 to 114 ± 8 nm for gradient copolymers. In more detail, DP_50_-based particles exhibit significant hydrophilic characteristics, close to water solubility, with measured sizes in the range of 115–140 µm and very low water contact angle (θ) values around 20°. For the other polymer particles, two distinct behaviors were observed depending on the monomer distribution. In the case of block-based particles, an increase in the DP leads to an increase in particle size (from 69 to 152 nm for DP_100_ and DP_200_), while gradient particles exhibit a size around 65–72 nm, remarkably displaying lower polydispersity indices compared to their block counterparts. Interestingly, for polymer particles with DP_100_, DP_150_, and DP_200_, an increase in chain length has little impact on their wettability, with θ values ranging from 53 to 65°. Furthermore, the particle glass transition temperature values follow the expected trend of increasing with chain length for both block and gradient particles. Recalling these results, it is possible to gain insight into the internal structure of polymer particles. Indeed, polymer nanoprecipitation occurs after the addition of a non-solvent to a polymer solution by a four-step mechanism: supersaturation, nucleation, condensation growth, and coagulation growth that leads to the formation of polymer particles [85]. Under such conditions, the essential driving force leading to the formation of polymer particles is clearly the hydrophobic effect between the PhOx monomers. To avoid considerable exposure of the hydrophobic residues in water, a strong self-association of the hydrophobic sections of the polymer chains occurs to form supramolecular assemblies. However, the presence of rigid aromatic phenyl rings leads to aromatic stacking of phenyl substituents with a shifted (or twisted) geometry relative to the face-to-face and/or T-shaped (or edge-to-face) orientation of the aromatics, thereby increasing the packing density of the polymer chains [86]. Therefore, we obtain core–shell polymer particles composed of a hydrophobic core containing PhOx monomers while the shell contains hydrophilic MeOx monomers. It is worth noting that this mechanism is valid for both block and gradient copolymers because for all copolymers, as the molar mass increases, a higher particle glass transition temperature value is observed due to the stronger π-stacking interactions [87]. Although these polymer particles, composed of a P(PhOx) core surrounded by P(MeOx) segments, are in dynamic equilibrium with their environment and the structural arrangement can be modified by environmental changes, the hydrophobic and aromatic stacking interactions prevent spatial changes and the dissociation of the supramolecular structure.

Since the polymer particles are open sponge-like colloidal structures capable of accommodating solvent(s) in both hydrophilic and hydrophobic regions, they can be used for the long-term stabilization of Pickering emulsions with emulgel properties using paraffin oil and isopropyl myristate [84]. These polymer particles effectively stabilize Pickering emulsions starting from 0.9 wt. % and allow to easily obtain high internal phase emulsions entirely based on biocompatible, low-toxicity, and tunable P(MeOx)-P(PhOx), opening the way to wide applications. Finally, it is noted that the other MeOx/PhOx ratio of 50/50 or 75/25 (*w*/*w*), whatever the chain length (degree of polymerization, DP = 50, 100, 150 or 200) and the monomer distribution (block or gradient), leads to classical emulsions without the formation of polymer particles [88]. This observation is clearly a consequence of the minimization of hydrophobic and stacking interactions (see above), proving once again the importance of intermolecular interactions, structural flexibility/rigidity, and fluidity/crystallinity.

#### 3.1.3. Surfactants

Surfactants are known to form micellar, lamellar, and vesicular structures. However, as previously discussed, these assemblies are soft and fluid because they are mainly driven by hydrophobic interactions. In contrast, the assembly of tectons using the concept of colloidal tectonics forms rigid and/or crystalline architectures driven by a combination of hydrophobic interactions, H-bonds, and electrostatic forces. Therefore, in order to obtain faceted structures, it is necessary to increase the rigidity and crystallinity of surfactant assemblies by additional strong and directional in-plane attractions (e.g., H-bond and/or electrostatic) and out-of-plane repulsions (e.g., electrostatic or steric).

**Figure 10:**
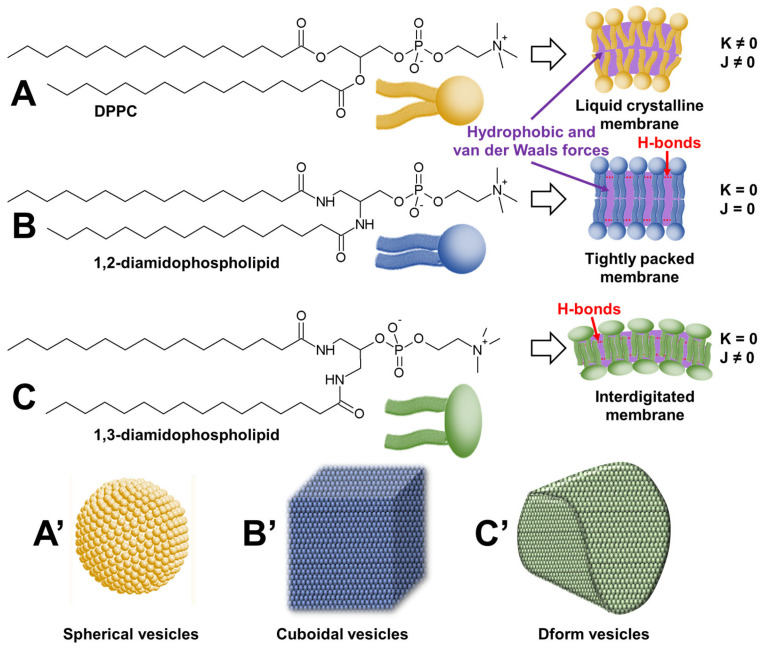
Structures of 1,2-dipalmitoylphosphatidylcholine (DPPC) (**A**), 1,2-diamidophospholipid (**B**), and 1,3-diamidophospholipid (**C**) and their self-assembly in spherical (**A’**), cuboidal (**B’**), and Dform (**C’**) vesicles. The figure highlights the dependence of the geometric shape of liposomes on the intrinsic Gaussian and extrinsic total curvature (K and J, respectively).

Faceted vesicles (i.e., rigid non-spherical liposomes) can be obtained by enhancing the crystalline characteristic of membranes. One way to achieve this is to replace the ester bonds of natural phospholipids with artificial amides that stabilize the interfacial region by H-bonds. In this regard, Neuhaus and coworkers used an artificial amide (1,2-diamidophospholipid) homologue of 1,2-dipalmitoylphosphatidylcholine (DPPC) to form cuboidal vesicles without using a template (Figure 10) [89].

Interestingly, vesicles are typically spherical to minimize surface tension, e.g., DPPC forms spherical vesicles. The authors use complementary H-bonds to increase the interactions between phospholipids. Recalling that DPPC forms only slightly faceted vesicles in the gel phase and that the fishbone packing of the lipids is not found in DPPC vesicles, the authors argue that the H-bond network present between 1,2-diamidophospholipid molecules leads to a minimization of the intermolecular distance and thus a maximization of the chain–chain attractions (i.e., dispersion forces). Consequently, intermolecular H-bonds between amide groups in 1,2-diamidophospholipids lead to planar bilayers with exceptionally tight packing, where the molecules are packed in a fishbone pattern as suggested by wide-angle X-ray scattering measurements. In practice, these rigid bilayers must be heated above their melting temperature to obtain fluid membranes, leading to vesicles. When cooled below their melting temperature, phospholipid vesicles give rise to structures that maximize flat surfaces and minimize edges, i.e., cuboidal structures. The authors propose to use these cuboid vesicles as drug delivery devices.

However, other fascinating structures can be obtained. For instance, the same team reported that 1,3-diamidophospholipids form faceted vesicles [90]. In these vesicles, the 1,3-diamidophospholipid molecules take a rigid interdigitated arrangement inside the bilayer membrane. Indeed, the thickness of the bilayer is comparable to the length of a single acyl chain of the phospholipid [91]. This tight packing results in the absence of spontaneous curvature, leading to faceted tetrahedral vesicles called Dform because of their resemblance to the letter D (see Figure 10). Since these vesicles are stable in a salt-containing buffer and under static conditions, Zumbuehl et al. investigated their use as drug delivery devices [92]. This study suggests that such loaded nanocontainers could potentially be used to treat atherosclerotic patients, as the drug is preferentially released in constricted vessels where shear stress is high [93].

The two previous examples are very instructive because they highlight the importance of molecular morphology on the shape of the supramolecular object obtained. Indeed, 1,2- and 1,3-diamidophospholipids are positional isomers capable of forming intermolecular H-bonds between the amide groups, leading to a maximization of chain–chain attractions (i.e., dispersion forces). The maximization of chain–chain attractions is optimal between 1,2-diamidophospholipid molecules due to the proximity of the two alkyl chains, which are spatially close. This effect is less for 1,3-diamidophospholipids, where the two alkyl chains are further apart, hence the need for chain–chain interdigitation within the bilayer membrane, suggesting that interdigitation is a major contributor to the formation of D-form vesicles. Therefore, in both cases, as the vesicles were formed from a single type of phospholipid, all vesicles are spherical above the melting temperature of the phospholipids because the liquid crystalline phase imposes no constraints on the geometry of the vesicles. However, upon cooling below the melting temperature, the intrinsic Gaussian curvatures and the extrinsic total curvatures of the bilayers (K and J, respectively) force the spheres to adopt alternative shapes with a clear dependence on the geometric shape of the liposomes (see Figure 10). Therefore, 1,3-diamidophospholipid forms rigid interdigitated bilayers and thus D-shaped vesicles (K = 0 and J ≠ 0). However, changing the phospholipid substitution pattern from 1,3 to 1,2 does not allow membrane interdigitation but preserves the H-bond network between phospholipid molecules, leading to cuboidal structures (K = 0 and J = 0). For DPPC, in the absence of an H-bond network, only spherical liposomes are observed (K ≠ 0 and J ≠ 0).

**Figure 11:**
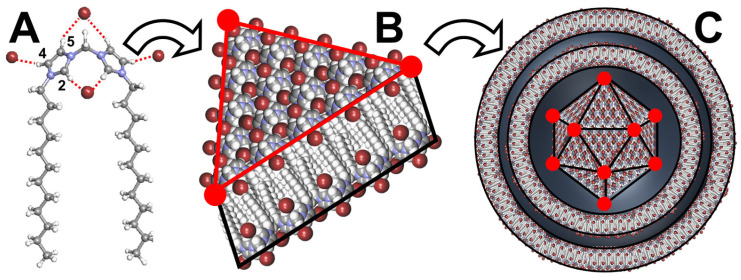
Structure of *N*,*N′*-didodecylmethylenediimidazolium ditriflate, schematic representation of H-bonds between imidazolium cations and anions (brown sphere (**A**)), planar bilayer arrangement with interdigitation of the akyl chains (**B**), and resulting vesicle with polyhedron core and spherical multilayer coat (**C**).

Polyhedral capsid-like cationic vesicles have also been observed. For instance, Pardin and his collaborators obtained this arrangement by the self-assembly of *N*,*N′*-dialkylmethylenediimidazolium ditriflate (Figure 11) [94]. Although there is no explanation for this extreme faceting of the vesicles in the original publication, it is now possible to propose an explanation based on current knowledge.

Indeed, it is known that imidazolium cations and anions are connected to each other to form H-bonded networks. In fact, the imidazolium ring serves as an H-bond donor through the H2 position and to a lesser extent through the H4 and H5 positions [95]. These large H-bond networks contribute to forming rigid membranes. In addition, the alkyl chains are spaced apart, leading to the intercalation of membrane sheets. Under such conditions, self-assembly into a closed 3D structure leads to a minimization of membrane intersections (edges) and a maximization of planar membrane faces, leading to a polyhedral vesicle. Recalling that the polyhedron core is surrounded by spherical multilayers, it is argued that spheres are produced to minimize the surface tension around the polyhedron core. Therefore, this arrangement resembles viruses: a polyhedron core surrounded by spherical layers. It is noteworthy that the polyhedron core depends on the length of the alkyl chain and the anion used. For instance, the dodecyl chain requires triflate anions to form polyhedrons while the hexadecyl chain requires bromides. These vesicles are used to entrap linear double-stranded (ds) DNA to protect it from enzymatic cleavage [95].

#### 3.1.4. Other Systems

In 2004, Valéry and his collaborators reported that octapeptides (acetate salts of cyclic Laureotide of sequence NH_2_-(D)Naph-Cys-Tyr-(D)Trp-Lys-Val-Cys-Thr-CONH_2_ and its cyclic derivative of sequence NH_2_-(D)Naph-Cys-Tyr-(D)Phe-Lys-Val-Cys-Thr-CONH_2_) self-assemble into nanotubes in water. The nanotubes are arranged in hexagons (with a packing parameter of 365 Å) and are highly monodisperse [96]. The tube diameter and wall thickness are 244 and 18 Å, respectively. Furthermore, the tube diameter is tunable by modifications of the molecular structure. The self-assembly of the nanotubes is due to the association of amphiphilic β-sheets and a systematic segregation of aromatic/aliphatic side chains. The same year, Hill et al. reported that amphiphilic hexa-*peri*-hexabenzocoronene molecules self-assemble to form nanotubular objects [97]. These objects are uniform with a 14 nm wide open hollow space and a 3 nm thick wall. The wall consists of helical arrays of 13 fused benzene rings stacking via π-interactions. The inner and outer surfaces are covered with hydrophilic triethylene glycol chains since the hexa-*peri*-hexabenzocoronene molecules form interdigitated bilayers across the alkyl chains. In a water–THF mixture, a helical coil is formed by the loose winding of the bilayer band, while in THF, the nanotubes are formed by the tight winding of the bilayer band.

### 3.2. Multi-Component Colloidal Systems

To achieve the formation of self-assembled (supra) colloidal structures from several building blocks, tectons must meet both structural and energy criteria. Indeed, it must be kept in mind that complementary tectons must, on the one hand, recognize each other and thus generate the seed, and on the other hand, allow the growth that leads to the formation of a 3D particle. This assembly process is mainly driven by solvophobic interactions and also by other complementary interactions such as H-bonds, aromatic stacking, electrostatic, or van der Waals forces, etc. (see above). These latter interactions are crucial to managing the appropriate structural flexibility/rigidity and fluidity/crystallinity needed to limit the formation of dynamic systems characterized by the exchange of molecules leaving and joining the aggregate (i.e., to avoid the reversible change in the number of tectons and the overall morphology in the resulting colloidal structure). Indeed, the tecton must fulfill both structural (recognition) and energetic criteria (tecton–tecton interactions must be higher than tecton–solvent interactions) [15]. In the search for synthetic systems reproducing the morphology, rigidity, and crystallinity of protein assemblies of all living organisms (see above), several strategies have been developed over the last two decades. Some of them are described in detail in the following sections depending on the origin of the architectures (i.e., organic or hybrid) and their applications if available.

#### 3.2.1. Organic Architectures

(*i*) *Cyclodextrin-based architectures*

**Figure 12:**
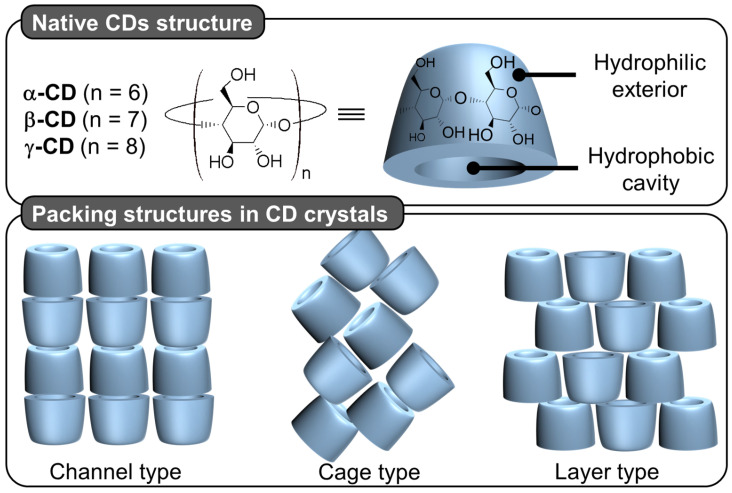
Native cyclodextrin (CD) chemical structure and schematic representation of packing structures of channel, cage, and layer type CD crystals.

Native cyclodextrins (CDs) are macrocyclic oligosaccharides, typically consisting of 6 (α-CD), 7 (β-CD), or 8 (γ-CD) glucose units linked by α-1,4 glycosidic bonds (Figure 12).

Morphologically, CDs have a truncated cone shape (i.e., bowl-shaped without a bottom) and are water-soluble but not in typical organic solvents [98]. However, their cavities are considerably less hydrophilic than the aqueous medium and are therefore capable of forming inclusion complexes with hydrophobic molecules. Since the rims are covered with primary and secondary hydroxyl groups, H-bonds are formed with water molecules, explaining their solubility in water [98]. In the crystalline state, CD inclusion complexes adopt three types of assembly modes: channel, cage, and layer types. Channel-type assembly, commonly observed with polymers, forms CDs stacked like coins in a roll [99]. In this mode, CDs can be arranged head-to-head or head-to-tail by H-bonding between the hydroxyl groups of neighboring CDs. On the other hand, cage architecture is observed when CDs are packed in a herringbone pattern [99]. In the latter mode, CDs are arranged side-by-side, forming layers offset by about half a CD [99]. With the smallest native CD (i.e., α-CD), cage-like structures are formed with small guests, whereas long or ionic guests induce channel-like structures. Channel types are generally preferred with the β- and γ-CDs. This diversity of assembly can be very useful in producing various colloidal systems, including nanoparticles, crystallites, lamellae, helical tubes, and polyhedrons (see below).

**Figure 13:**
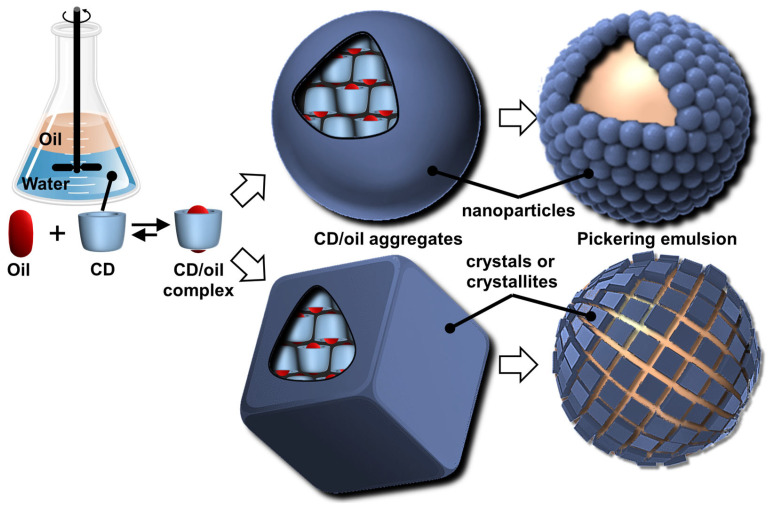
Schematic representation of sequential self-assembly of complementary tectons (CDs and oil molecules), leading to Pickering emulsions.

Particles or crystallites can be easily obtained using native CDs and various non-charged guests. Indeed, mixing CDs in biphasic oil/water systems gives oil-in-water Pickering-like emulsions stabilized by partial wettable insoluble CD/oil inclusion complexes [100]. As the colloidal structure depends on the experimental conditions, the nature of the precipitated fraction can be crystals, crystallites, or spherical nanoparticles [15,101,102,103]. In detail, these systems result from the formation of inclusion complexes between CDs and oil molecules, leading to the formation of small nuclei in the dispersion; the nuclei then grow as insoluble inclusion complexes from the liquid attached to them. These steps allow the formation of a 3D structure by self-assembly or self-organization within the biphasic mixture, leading to crystallites or nanoparticles depending on the experimental conditions and the guest structure (Figure 13) [15].

In 2024, Hou and Xu revealed the role of oils in the formation of CD/oil inclusion complex crystallites using different oils (e.g., linear alkanes, oleic acid, glycerol trioleate, and soybean oil). The authors observed that the inclusion complexes tend to grow in clusters and terminate at a certain finite size as long columns or lamella plates with well-defined facets, depending on subtle changes in the molecular architecture of the oil guests used [104]. Similar to the crystal structures of host–guest complexes, these assemblies are expected to contain channel, cage, and/or layer-like packing structures through the formation of a network of H-bonds between CDs (see above). The replacement of native CDs with chemically modified CDs alters the solubility of the inclusion complexes and renders them unable to assemble, highlighting the importance of H-bonds between CDs within the edifices. However, the growth of “particles” is limited due to the increase in interfacial rigidity with particle emergence, resulting in slower CD or oil transfer rates across the L/S/L interface [15]. Furthermore, the growth of these colloidal assemblies is also inhibited by steric repulsions.

Due to the virtually infinite number of systems that can be obtained by simple guest exchange, many applications have been considered. First, Pickering emulsions based on the extemporaneous formation and adsorption of insoluble CD/oil complexes at the water/oil interface are very interesting for performing catalytic reactions. One such system uses [Na]_3_[PW_12_O_40_] as a water-soluble catalyst to perform the oxidation of olefins, organosulfides, and alcohols in the presence of hydrogen peroxide as an oxidant [103]. These heptane-in-water Pickering catalytic emulsions are very efficient reaction media, e.g., the epoxidation of cyclooctene proceeds at a competitive rate (370 h^−1^) with a good yield (>99% in 30 min) and a high selectivity (>99%). These good results can be attributed to the privileged interfacial contact between the substrate and the catalyst. Furthermore, phase separation is achieved simply by centrifugation or heating, and a catalytic system based on colloidal tectonics is highly compatible with some “green chemistry” concepts because these systems can be used without any organic hazardous solvents for liquid substrates [103]. Secondly, since biocidal phytochemicals (e.g., carvacrol and terpinen-4-ol) can be used as oil phases, the development of surfactant-free and silica-free phytochemical- and β-CD-based self-assembled Pickering emulsions was considered to potentiate the antimicrobial and antibiofilm activity of miconazoctylium bromide [102]. The results clearly show that the emulsion containing carvacrol and miconazoctylium bromide exhibits synergistic effects against fungi, additive responses against bacteria, and very high activity against methicillin-resistant *S. aureus* biofilms. In order to obtain fully bio-based antimicrobial Pickering emulsions, petroleum-based miconazoctylium bromide is replaced by undecylenic acid [105]. However, this castor oil derivative, already used as a bio-based drug to treat fungal infections, is less effective than petroleum-based drugs. The carvacrol-based emulsion is +390% and +165% more active against methicillin-resistant S. aureus compared to commercial undecylenic acid and azole emulsions. In addition, this emulsion is highly effective against *C. albicans* (up to +480% more potent than commercial undecylenic acid ointment). This eco-friendly emulsion also shows remarkable activity against *E. coli* and methicillin-resistant *S. aureus* biofilms.

In addition, CD-based Pickering emulsions allow the production of other derivative systems such as cyclodextrinosomes or beads (i.e., microcapsules) [106,107]. For instance, Mathapa and Paunov used oil-in-water Pickering emulsions (oil = *n*-tetradecane, tricaprylin, isopropyl myristate, sunflower, or silicone) stabilized by microrods and microplatelets of CD/oil inclusion complexes [106]. As these microcrystals remain irreversibly anchored at the droplet interface, these emulsions can serve as a model for the preparation of cyclodextrinosomes obtained solely by the assembly of CD/oil inclusion complexes, leading to a crystalline phase on the surface of the droplets that retains its stability after removal of the solvents. As the authors point out, cyclodextrinosomes can be used in cosmetics, personal and household care products, and pharmaceutical formulations. A very close system relies on the interactions between CDs and vegetable oils to produce CD-based beads [107]. By adding vegetable oil to an aqueous solution of CD, it is possible to obtain biphasic systems separated by an interfacial film consisting of triglyceride/CD inclusion complexes. By stirring, the authors obtained oil-in-water emulsions, leading to the crystallization of inclusion complexes. After a few days of continuous stirring, an aqueous suspension of CD-based beads is obtained [108]. X-ray diffraction studies reveal that the beads exhibit a crystalline organization, and microscopic analyses show that their internal structure consists of a matrix containing numerous oil compartments [107]. Indeed, unlike cyclodextrinosomes, beads are obtained after a few days of continuous agitation, with oil droplets interconnected via the fusion of crystalline zones, leading to droplets dispersed in a spherical crystalline matrix of CD/oil inclusion complexes [109,110,111]. Thus, regardless of their preparation, cyclodextrinosomes and beads refer to similar structures emerging from the packing of nanoparticles via a bottom-up construction.

**Figure 14:**
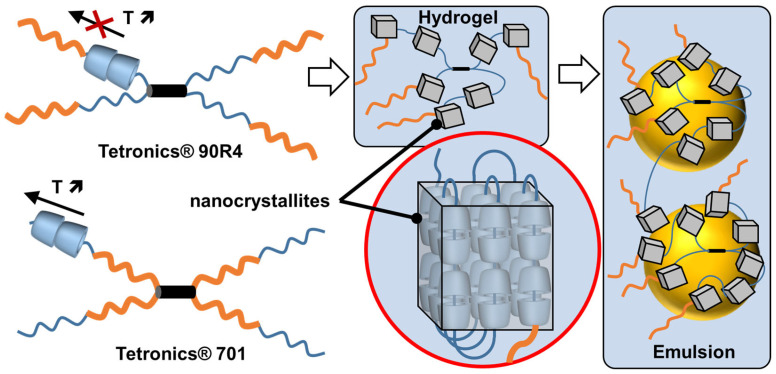
Schematic representation of Tetronics^®^ 90R4 and 701 (orange = polypropylene oxide block and blue = polyethylene oxide block, black cylinder = central ethylene diamine), structure of α-CD/Tetronics^®^ nanocrystallites and resulting Pickering emulsion.

If guest polymers are used, CD inclusion complexes form channels containing the guest polymers (see above). For instance, the formation of inclusion complexes between high-molecular-weight polyethylene glycol (PEG 20,000 or 35,000 g/mol) and native α-CDs, leading to the formation of supramolecular hydrogels after heating/cooling cycles. Indeed, the columnar CD domains (nanocrystallites), formed by nucleation and growth processes, act as physical cross-links alongside the non-included polymer chains (i.e., polypseudorotaxanes) [112]. However, crystallite growth is limited by the diffusion rate at which α-CDs are transferred from the aqueous bulk to the growing crystallites. After the addition of oil and stirring, these hydrogels allow the formation of oil-in-water Pickering emulsions. These emulsions are used to perform the biphasic catalytic hydroformylation of higher olefins. Under these conditions, the mass transfer is drastically enhanced in comparison to pure biphasic conditions or in the presence of α-CD or PEG alone due to the enhanced contact between the organic substrate and the rhodium catalyst associated with trisulfonated triphenylphosphine as a water-soluble ligand. The catalytic performance of these Pickering emulsions can be improved by adding randomly methylated β-CD (RAME-β-CD) [113]. Indeed, the catalytic activity is increased because this CD acts as a supramolecular carrier capable of transporting hydrophobic olefins. Unfortunately, an excess of RAME-β-CD leads to lower catalytic performance due to the instability of Pickering emulsions. Finally, to improve the thermal stability of the catalytic system, poloxamines (Tetronics^®^, a class of polyethylene oxide (PEO)/polypropylene oxide (PPO)-based amphiphiles linked to a central ethylene diamine moiety by way of the nitrogen atoms) were used instead of PEG [114]. The results reveal that the catalytic systems using reverse sequential Tetronic^®^ 90R4 are more efficient than those containing conventional sequential Tetronic^®^ 701 (Figure 14). As in reverse poloxamines (Tetronics^®^ 90R4), the PPO segments are placed at the periphery, the PPO blocks act as “stoppers”. Indeed, the α-CDs are kinetically trapped since the ends of the system are larger than the internal PEOm which prevents the dissociation (scrolling) of the α-CDs. The opposite is true for classical sequential poloxamines (Tetronic^®^ 701), where the α-CDs can be displaced upon heating (see Figure 14). Consequently, α-CD/Tetronics^®^ 90R4 nanocrystallites exhibit superior thermal stability compared to α-CD/Tetronic^®^ 701. At 80 °C, the stability of these α-CD/Tetronics^®^ 90R4-based emulsions is superior to that of α-CD/Tetronics^®^ 701, leading to olefin conversion at competitive reaction rates due to enhanced mass transfer.

The previously reported α-CD/PEG-based colloidal tectonics system can be used as a common base for formulation, leading to a range of dosage forms (i.e., hydrogels, Pickering emulsions, and cyclodextrinosomes) [16]. Stable ethanol-free hydrogels can be prepared by mixing, in water, α-CD, PEG, and antifungal drugs (miconazole, miconazole nitrate, or econazole nitrate) as inclusion complexes with 2-hydroxypropyl-β-CD to increase the solubility of the drugs in the hydrogels. As previously described, oil-in-water Pickering emulsions can be obtained from unloaded gels by adding paraffin oil containing antifungals in their native form. Once dried, the emulsions provide loaded cyclodextrinosomes. The properties are significantly improved compared to commercially available formulations in terms of stability, with even a marked improvement in antimicrobial activity (up to 1.6). These dosage forms avoid the use of petro-sourced surfactants or modified silica nanoparticles, providing solutions to the current trend of simplification of formulas in terms of ingredients.

**Figure 15:**
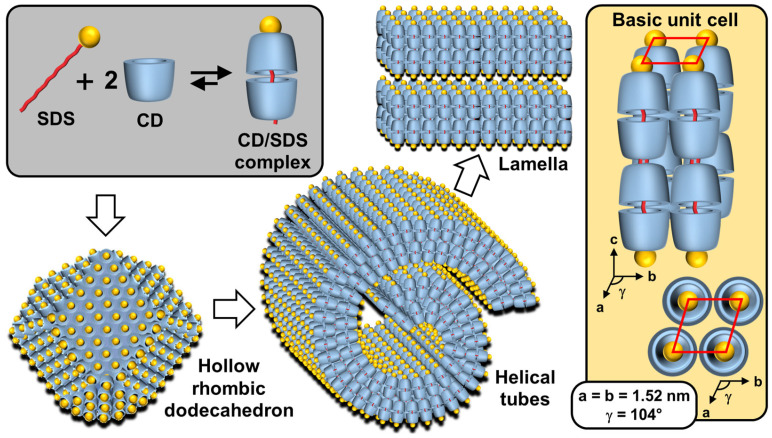
Schematic representation of self-assembled β-CD/SDS inclusion complexes (2:1 stoichiometry) into lamellar, helical tubular, and hollow rhombic dodecahedral architectures. The right inset shows the basic unit cell of columnar inclusion complexes in a 2D rhombic packing (quasi-monoclinic with the following parameters a = b ≠ c and α = β = 90° and γ ≠ 90°).

If ionic guests are used, CD inclusion complexes preferentially form channels containing the guests (see above). When ionic guest molecules are surfactants, CDs disrupt the self-assembly properties of these last by sequestrating their hydrophobic regions. For instance, the complexation of β-CD with sodium dodecyl sulfate (SDS) leads to the formation of 1:1 and 2:1 inclusion complexes [115]. However, inclusion complexes, depending on the sample concentration and temperature, tend to organize into aggregates with an ordered structure. To mimic proteins that can readily assemble into rigid and crystalline structures such as viral capsids (see above), Jiang and coworkers reported the use of β-CD/SDS inclusion complexes (2:1 stoichiometry) capable of self-assembling into a variety of structures such as lamellar, spiral wound bilayers (multilamellar tubes), and hollow rhombic dodecahedral architectures (Figure 15) [116,117,118].

The nature of architectures directly depends on the concentration of β-CD/SDS inclusion complexes, e.g., rhombic dodecahedra are obtained in the range of 4–6 wt. % while helical tubes and lamellae are observed, respectively, between 6–25 and 25–50 wt. %. In detail, the rhombic dodecahedral geometry (~1 µm) is hollow and has sharp edges as revealed by transmission electron microscope and atomic force microscope results. Unlike viral capsids where an icosahedral geometry is classically observed, the dodecahedral architecture is dominant in this case due to the 2D rhombic basic unit cell (see below and Figure 15).

Furthermore, it is worth noting that the size of the hollow rhombic dodecahedron is significantly larger than that of classical icosahedral viruses. Helical tubes consist of a bilayer wound with open ends. They are large (average length about 40 µm) and rigid. The diameter is monodisperse around 1 µm. The persistence length of the helical tubes is at least of the order of millimeters. The distance between the walls is large due to charge repulsion and depends on the condensation of cations. In addition, dilution causes swelling of the structure due to the penetration of water molecules into the interwall space. The bilayers are extremely rigid and planar due to their crystalline nature. The distance between two bilayers depends on the effective charge, with potential charge screening by counterions.

From a general point of view, the formation mechanism of these ordered architectures relies on CD/surfactant inclusion complexes as primitive building blocks. The self-assembly of these inclusion complexes is driven by strong direct and indirect (water-mediated) H-bonds, leading to channel-like arrangements, regardless of the size of the CD cavity. However, α-CD, β-CD, and γ-CD/surfactant form crystal structures with a hexagonal, rhombic, and square arrangement of channel-type inclusion complexes in the 2D lattice [119]. Therefore, β-CD/SDS inclusion complexes, with a 2:1 stoichiometry, form colloidal stable suspensions, presenting a crystalline substructure made from the primitive building blocks (i.e., the β-CD/SDS inclusion complexes). Since the basic unit consists of two inclusion complexes where the β-CDs are in a channel-type arrangement with a rhombic packing (see Figure 15), the basic unit grows into lamellae, spiral wound bilayers (multilamellar tubes), and/or folds into polyhedrons, depending on the concentration and temperature. This growth is driven by strong direct and indirect (water-mediated) H-bonds [116]. The importance of these H-bonds is confirmed by the fact that chemical substitutions on the hydroxyl groups of CDs and the use of chaotropic additives (disturbing the H-bonds) depress the assemblies. In addition, temperature is also a key parameter, as it weakens the H-bond network connecting the CDs, leading to structural changes in the observed structures. The bilayer-like assembly is determined by three factors: (i) the high rigidity of the membranes due to the H-bonds between CDs, (ii) the electrostatic repulsions between the faces due to the anionic nature of SDS, and (iii) the surface tension on the edges of the bilayer membrane that leads to the curvature of the edges and the formation of a closed 3D structure.

A clear similarity can be established between the colloidal architectures observed for β-CD/SDS inclusion complexes and protein self-assembly (see above). For example, capsid proteins are held together by specific directional interactions between the different protein units and by nonspecific, mainly electrostatic, interactions with the genetic material (see above). These supramolecular colloidal assemblies of β-CD and SDS exhibit characteristic sizes ranging from a few nanometers for a single primitive building block to several micrometers. Electrostatic interactions and H-bonds are identified as the driving forces for the self-assembly of β-CD/SDS inclusion complexes. The morphologies of the aggregates could be reversibly controlled using temperature or concentration changes.

**Figure 16:**
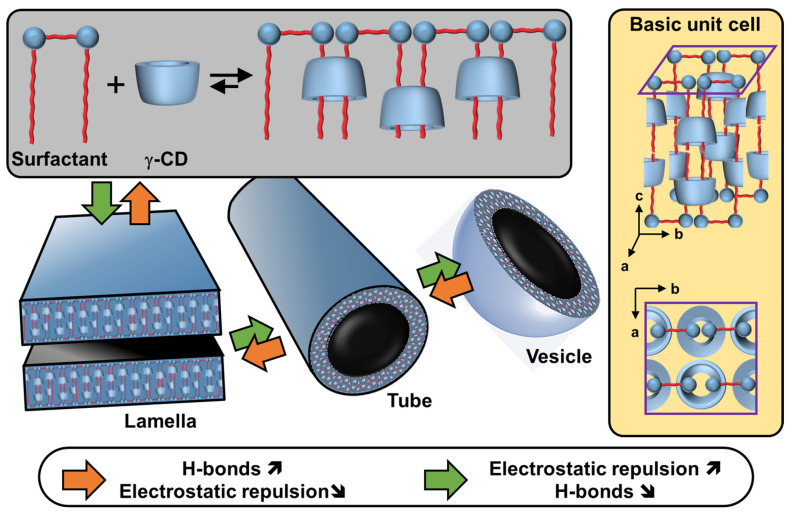
Schematic representation of self-assembled γ-CD/*N*,*N′*-didodecyl-*N*,*N*,*N′*,*N′*-tetramethyl-*N*,*N′*-hexamethylenediamines inclusion complexes into lamellar, tubular, and spherical vesicular architectures. The right inset shows the basic unit cell of columnar inclusion complexes in a tetragonal packing.

In 2023, Liu et al. reported that polymer-like building blocks consisting of inclusion complexes between γ-CDs and *N*,*N′*-didodecyl-*N*,*N*,*N′*,*N′*-tetramethyl-*N*,*N′*-hexamethylenediamine surfactants exhibit various colloidal structures such as vesicles, nanotubes, and sheets in aqueous solutions with increasing concentrations (Figure 16) [120]. A single tetragonal unit with a 1:1 γ-CD/surfactant ratio is obtained by host–guest interactions (see Figure 16). This tetragonal unit enables the growth of bilayer sheets due to H-bonding and electrostatic interactions. However, the morphologies of the bilayer architectures are multi-responsive to stimuli such as temperature, solvent additives, and ions. Indeed, the decrease in H-bonds (i.e., increased in-plane fluidity) and the increase in out-of-plane electrostatic repulsions (i.e., increased repulsions between surfactant head groups) enable a transition from bilayers to nanotubes and then from tubes to spherical vesicles. As these transitions are reversible, this system has promising potential for drug delivery applications in the future. Due to the great interest in these systems, many studies have been conducted on them [121,122,123,124].

(*ii*) *Catanionic surfactants-based architectures*

**Figure 17:**
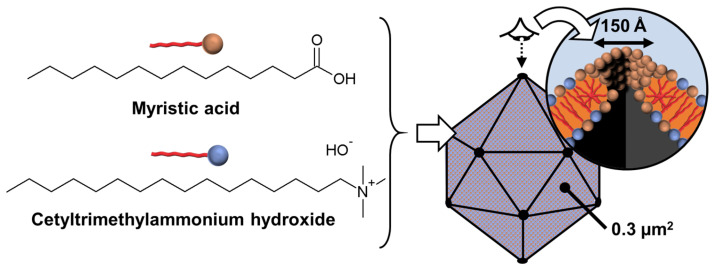
Schematic representation of self-assembled catanionic mixtures into icosahedral architectures made of about 10^6^ ion pairs. The right inset shows one of the twelve pores produced by about 200 molecules due to the partial segregation of the anionic surfactant in excess.

Catanionic mixtures contain both anionic and cationic surfactants, where one ion acts as a counterion of the other. Under appropriate conditions, these catanionic mixtures have been shown to successfully mimic the morphologies of protein assemblies. These systems, which can self-assemble into regular hollow icosahedral shapes similar to those observed for viral capsid proteins (see above), have been extensively studied by Zemb and colleagues. The first report on close-packed icosahedra made from synthetic amphiphiles was published in 2001 [125]. The authors reported that salt-free mixtures of myristic acid and cetyltrimethylammonium hydroxide can self-assemble into hollow aggregates with a regular icosahedral shape for mole fractions between 0.5 and 0.75 and for a total surfactant volume fraction <10^−3^ (Figure 17). However, some steps are required compared to previous systems. For instance, the solution of the two surfactants, where the anionic component (i.e., the myristic acid) is in excess, is first heated to 60 °C for a few minutes and then cooled to room temperature under constant stirring until micrometer-sized icosahedrons form in the fluid supernatant (phase separation leading to icosahedrons can be accelerated by gentle centrifugation at 3000× *g*).

Recalling that mild temperature cycling (i.e., heating to ensure redissolution and cooling to restore icosahedrons) and slow dissolution do not involve significant energy input other than the dispersion of colloidal objects, the authors argue that icosahedrons are maintained solely by molecular interactions. Indeed, these icosahedral aggregates are stabilized by the crystallization of surfactant alkyl chains. The chain melting transition is mole fraction-dependent and lies in the range of 50–65 °C according to differential scanning calorimetry. The icosahedral structures have a size of about 1 µm and a mass of ~1010 Da. As with β-CD/SDS systems, their sizes are larger than those of all known icosahedral viral capsids. Due to the excess of myristic acid, the electric charge of the icosahedrons is negative. In addition, pores, located at the vertices of the icosahedrons, are observed on freeze-fractured samples due to the excess of anionic surfactant molecules that cover the edges of the pores and form half-micelles (see Figure 17). Unfortunately, the icosahedrons are metastable, meaning that after several months, phase separation occurs. However, the addition of glycerol somewhat prolongs their stability. If the excess component is insufficient to form twelve pores per vesicle, this leads to the formation of nanodiscs or large perforated crystalline bilayers. In 2004, Zemb and his collaborators proposed a general mechanism explaining how the ratio of cationic and anionic surfactants controls the shape of the resulting crystallized colloidal structure [126]. The shapes of the aggregates are determined solely by the initial molar ratio. Indeed, molecular segregation occurs, leading to accumulation on the edges or pores of excess surfactant instead of being incorporated into the crystalline bilayers. As these icosahedral structures combine wall rigidity and vertex holes, they may be useful for the controlled release of drugs or DNA with probable functional superiority over soft vesicles obtained from classical surfactant systems.

Non-spherical structures can be generated from the self-assembly of unequal charged anionic and cationic amphiphiles, leading to small curved vesicles presenting flat ionic domains on the vesicle surface due to the interplay of electrostatic, hydrophobic, and steric forces. For instance, Greenfield et al. utilized the strong electrostatic interaction between the head groups of trivalent cationic and monovalent anionic surfactants to increase the cohesive energy of amphiphiles and promote the formation of faceted vesicles [127]. Faceted vesicles can also be obtained by in situ deprotonation of a surfactant. For instance, the mixture of perfluorononanoic acid (PFNA) or perfluorodecanoic acid (PFDA) with NaOH leads to polyhedral vesicles formed by PFNA/PFN^−^ or PFDA/PFD^−^ surfactants [128]. The PFDA/NaOH system is pH-sensitive as revealed by freeze-fracture transmission electron microscopy images where a transition from the faceted vesicle phase to the sponge phase (i.e., L_3_ phase) is observed. It is noteworthy that this transition is not observed for the PFDA/NaOH system. The mechanism leading to the formation of faceted vesicles and the mechanism of transition from vesicles to perforated lamellae and faceted vesicles have been widely discussed and modeled in the literature [129,130,131].

#### 3.2.2. Inorganic–Organic Hybrid Architectures

**Figure 18:**
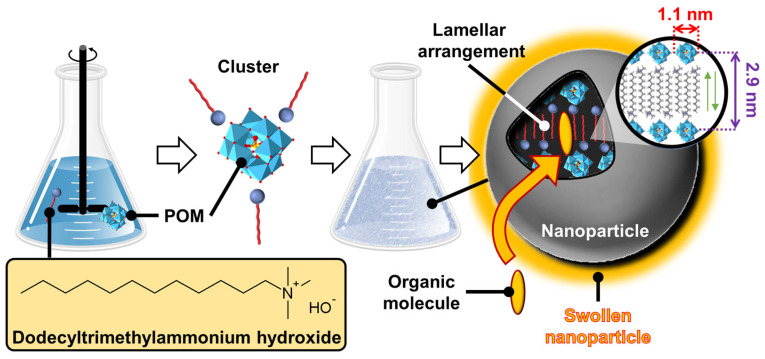
Schematic representation of self-assembled mixtures into inorganic–organic hybrid nanoparticle architectures made of H_3_PW_12_O_40_ (1 equiv) and dodecyltrimethylammonium hydroxide (3 equiv). The lamellar internal arrangement allows the incorporation of small organic molecules, leading to swollen nanoparticles.

The fascinating structures obtained using the tectonic colloidal approach can be used to obtain supramolecular hybrid functional architectures. In 2012, the colloidal engineering of novel hybrid nanoparticles was reported [132]. This system results from the ionic metathesis between negatively charged polyoxometalates (POMs) and cationic surfactants. These two “hydrophilic” and “hydrophobic” tectons lead to the formation of uncharged clusters. Indeed, in an aqueous solution, the neutralization of H_3_PW_12_O_40_ (1 equiv) with dodecyltrimethylammonium hydroxide, [C_12_][OH] (3 equiv) leads to the formation of hybrid clusters [C_12_]_3_[ PW_12_O_40_]. In order to decrease the hydrophobic/water contact, the clusters self-assemble to spontaneously form nanoparticles. Transmission electron microscopy and dynamic light scattering experiments reveal that the nanoparticles are monodisperse with a diameter of approximately 35 nm. The internal structure of the [C_12_]_3_[PW_12_O_40_] nanoparticles, studied by small-angle X-ray scattering measurements, reveals a lamellar arrangement. Indeed, these nanoparticles consist of parallel inorganic planes of [PW_12_O_40_] anions separated at a mesoscopic scale by organic bilayers of interdigitated cationic surfactant chains (Figure 18).

It should be noted that this arrangement is highly predictable as it is already observed in the literature in the solid state. For instance, surfactant/inorganic phases containing the adamantane thiogermanate anion [Ge_4_S_10_]^4−^, in combination with a stoichiometric amount of alkyltrimethylammonium cations, crystallize in the triclinic space group *P*-1 and contain parallel planes of [Ge_4_S_10_]^4−^, separated by interdigitated cationic surfactant bilayers [133]. As mentioned before, this self-assembly is governed by the nucleation and growth processes. However, during growth, some defects appear in the organization, leading to the appearance of charges [134]. The size or shape of the nanoparticles depends on the choice of POM. Indeed, various colloidal structures such as spherical, pseudospherical, and tubular structures can be obtained for [PW_12_O_40_]^3−^, [PW_11_VO_40_]^4−^, and [PW_9_V_3_O_40_]^6−^, respectively [135]. From TEM images, the size is estimated to be about 35, 60, and 392 nm for [C_12_]_3_[PW_12_O_40_], [C_12_]_4_[PW_11_VO_40_], and [C_12_]_6_[PW_9_V_3_O_40_], respectively [135]. Interestingly, however, internal lamellar packing is observed regardless of the size or shape of the POMs. Indeed, the lamellar organization results from the combined effects of hydrophobic, van der Waals, electrostatic, and steric interactions that allow the tectons to organize [135]. Like casein micelles, nanoparticles are porous due to their internal lamellar packing in which small organic molecules can be accommodated, leading to swollen particles (see Figure 18) [134]. Therefore, in the presence of water and aromatic oil, these nanoparticles can be used to stabilize water-in-oil Pickering emulsions consisting of shell-like architectures around the water droplets. These emulsions exhibit very high stability due to the strong cohesion between the particles located in the interfacial layer. Moreover, the nanoparticles are observed to interlock, which increases the interfacial elasticity due to the penetration of oil molecules into the nanoparticles [134]. Consequently, these emulsions offer a general route for the construction of colloidosomes [134].

**Figure 19:**
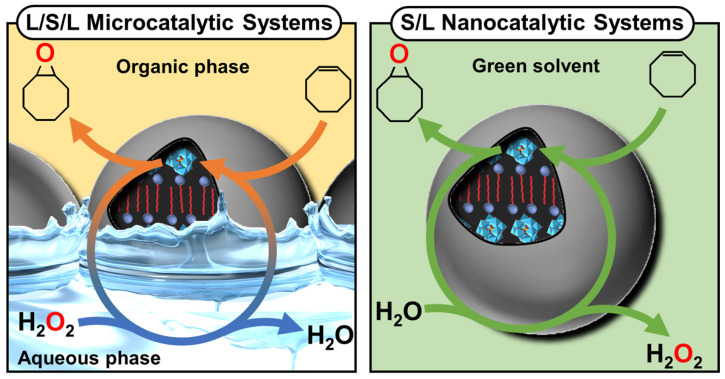
Schematic representation of the two catalytic systems using inorganic–organic hybrid nanoparticle architectures: Pickering emulsions (**left**) and dispersion (**right**).

As these Pickering emulsions, stabilized by inorganic–organic nanoparticles, exhibit high stability, a very large water/oil interface, and potential catalytic activity, their oxidation capabilities involving hydrogen peroxide as an oxidant were studied. Moreover, it is noted that although the emulsions exhibit long-term stability, the droplets break up and the final coalescence can be induced on demand by gentle centrifugation at 900× *g*, leading to three well-separated phases: water, oil, and hybrid nanoparticles. Two catalytic systems were used (Figure 19). The first, published in 2012, uses the [C_12_]_3_[PW_12_O_40_] hybrid nanoparticles to perform the epoxidation of olefins in a biphasic water/toluene system in the presence of hydrogen peroxide as an oxidant [132]. As expected, the quantitative epoxidation of various olefins (oct-1-ene, cyclohexene, cyclooctene, and limonene) is achieved with easy separation of the product and catalyst. For instance, the epoxidation of cyclooctene proceeds at a competitive rate (32.3 h^−1^), good yield (98% in 3 h), and high selectivity (>99%). This highly efficient reaction medium is due to the larger surface area of the water/oil interface where the catalytic nanoparticles are located. It is relevant to note that the catalytic performances are obtained under mild conditions (65 °C) and without stirring, indicating that the process is not limited by mass transfer. In other words, the reaction is solely driven by the catalytic cycle. The second catalytic system, published in 2014, uses [C_12_]_3_[PW_12_O_40_] nanoparticles to perform the epoxidation of olefins directly and only in eco-compatible solvents (e.g., cyclopentyl methyl ether, 2-methyl tetrahydrofuran, methyl acetate, and glycerol triacetate) [136]. Indeed, hybrid particles are able to form stable dispersions due to their amphiphilic properties. As for the previous system, cyclooctene epoxidation proceeds at competitive rates (initial turnover frequency, TOF_0_ > 260 h^−1^), with good yield (>95%) and high selectivity (>99%). It is worth noting that [C_12_]_3_[PW_12_O_40_] nanoparticles exhibit a catalytic activity 10 times higher than that obtained with the native catalyst ([Na]_3_[PW_12_O_40_]). This effect is attributed to the stable dispersion and the accommodation of cyclooctene molecules inside the porous nanoparticles. These two catalytic systems combine the advantages of heterogeneous and homogeneous catalysis, namely high activity and selectivity, easy phase separation, and catalyst reuse (after filtration and/or distillation).

**Figure 20:**
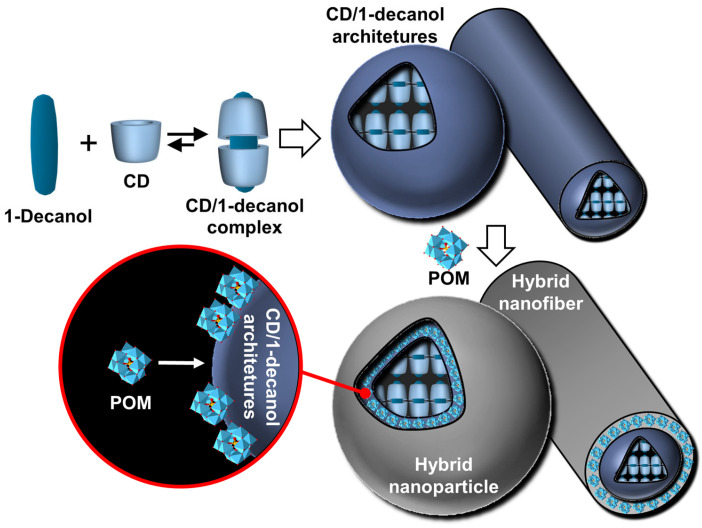
Schematic representation of the sequential synthesis of hybrid architectures by self-assembly of CD and 1-decanol followed by addition of POMs.

Another inorganic–organic hybrid system can be easily obtained by reusing the self-assembled β-CD/guest inclusion complexes in nanoparticle architectures (see above). Indeed, POM anions have a strong propensity to adsorb on electrically neutral surfaces due to their large, sticky, polarizable, and salting-in nature. For instance, Stoddart and co-workers demonstrated that complexation between CDs and POMs leads to the formation of inorganic/organic hybrid sandwich complexes where a POM anion is encapsulated by the primary faces of two CDs [137]. Similar observations were made by Bauduin et al., who reported that the adsorption of POMs on the surface of nonionic micelles was mainly due to the entropy gain caused by the release of several molecules of water of hydration in the bulk [138,139]. Indeed, the adsorption of POMs on the surface of micelles leads to a partial dehydration of the polar heads of surfactants and POMs. Similar effects can be invoked with CD particles (see above). Based on this behavior, Pacaud and his collaborators construct core–shell hybrid nanoparticles or nanofibers in a hierarchical manner by the sequential self-assembly of three complementary tectons: β-CD, 1-decanol, and POM (Figure 20) [140]. The following mechanism occurs: (i) β-CD/1-decanol inclusion complexes are formed, (ii) β-CD/1-decanol inclusion complexes self-assemble to yield nanoparticles, and (iii) the adsorption of POMs onto the polar neutral interface of CDs allows the formation of spherical core–shell hybrid nanoparticles (~40 nm) and nanofibers (15–25 nm in width and 7.5 µm in length).

**Figure 21:**
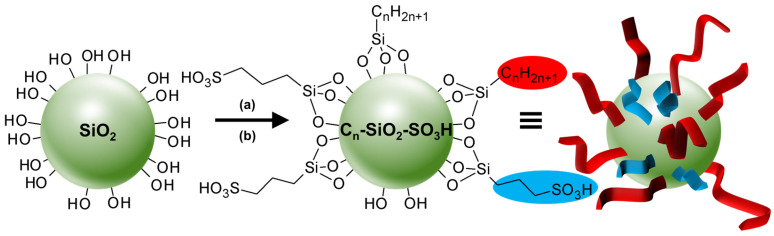
Synthesis of acidic/amphiphilic silica nanoparticles C_n_-SiO_2_-SO_3_H (n = 3, 8 or 18) and their schematic representation: (a) 1 g of Aerosil^®^ 200, 4 mmol of alkyltrimethoxysilane, 16 mmol of (3-mercaptopropyl)trimethoxysilane, H_2_O/EtOH pH 9.6, reflux, 24 h; (b) 60 mL H_2_O_2_ (50%), CH_3_CN, 40 °C, 24 h.

While it is possible to obtain hybrid nanoparticles using molecular tectons as in the two previous examples, it is also possible to directly influence the assembly of the particles. In this context, Yang and his collaborators used the colloidal tectonics approach to achieve tandem synergistic Pickering interfacial catalysis. This catalytic system is based on the use of two “surface-active” nanoparticles: [C_12_]_3_[PW_12_O_40_] (see above) and silica nanoparticles functionalized by alkyl chain groups and sulfonic acids (C_n_-SiO_2_-SO_3_H, see Figure 21).

**Figure 22:**
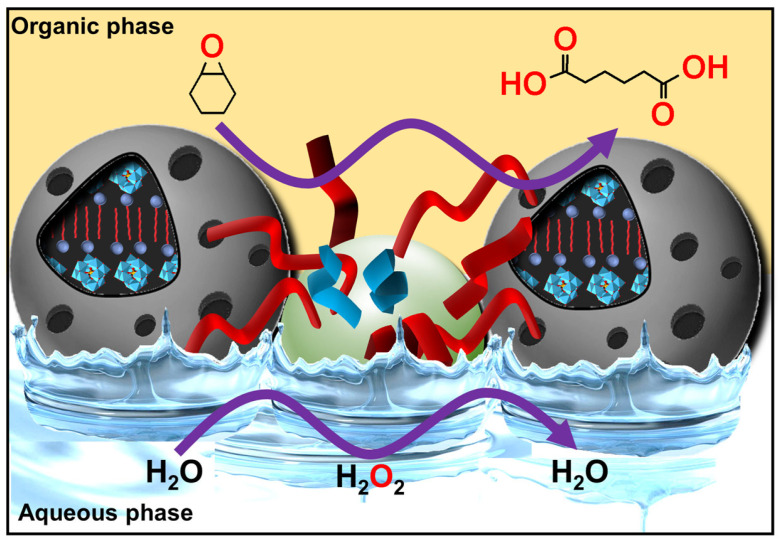
Schematic representation of oxidative cleavage of epoxycyclohexane in a water-in-toluene Pickering emulsion stabilized by [C_12_]_3_[PW_12_O_40_] and C_n_-SiO_2_-SO_3_H catalytic nanoparticles.

These nanoparticles, containing both recognition and catalytic sites, are used to control the formation and properties of Pickering emulsions, such as stability [141]. Due to the penetration of the alkyl chains of C_n_-SiO_2_-SO_3_H nanoparticles into the self-assembled hybrid, in [C_12_]_3_[PW_12_O_40_] nanoparticles that possess a porous structure composed of hydrophilic and lipophilic regions (see the internal arrangement of the particle, presented above), an interfacial self-assembly occurred, leading to the interlocking of the two nanoparticles. In a biphasic water/oil system, the two nanoparticles lead to elastic “springs” in the interfacial layer, giving rise to water-in-oil Pickering emulsions with long-term stabilities. These emulsions are used for the synthesis of adipic acid from the one-pot oxidative cleavage of epoxycyclohexane in the presence of aqueous hydrogen peroxide (Figure 22). It should be noted that, from an industrial point of view, adipic acid is the most important dicarboxylic acid (~2.5 billion kg/year) due to its use as a precursor for the synthesis of nylon. As expected, the catalytic performances were significantly enhanced due to the interfacial co-adsorption of the two nanoparticles catalyzing the hydrolysis and oxidation steps involved in this oxidative cleavage. For instance, under optimal conditions (80 °C, 500 rpm), the oxidative cleavage of epoxycyclohexane reached almost complete conversion (>99% after 12 h) and high selectivity (94%). To extend the scope of this system, the authors also studied the oxidative cleavage of cycloheptene, cyclooctene, 1-methylcyclohexene, and 4-methylcyclohexene oxides under the same reaction conditions. For all these substrates, high conversions and excellent selectivities are obtained. As with the previous Pickering catalytic system, the advantages of homogeneous (high activity and selectivity) and heterogeneous (simple phase separation and catalyst reuse) catalysis are combined. Moreover, the advantages of the colloidal tectonic approach, such as the flexibility and versatility of self-assembled systems, are also highlighted.

**Figure 23:**
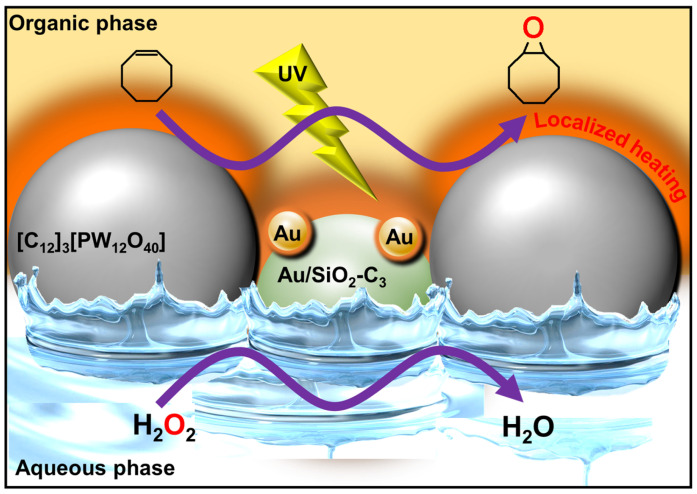
Schematic representation of epoxidation of cyclooctene in a water-in-toluene Pickering emulsion stabilized by [C_12_]_3_[PW_12_O_40_] and Au/SiO_2_-C_3_ nanoparticles acting, respectively, as catalyst and as on-site heater/plasmon activators under UV irradiation.

Using a similar approach, Feng et al. used a combination of plasmonic Au-loaded amphiphilic trimethoxy(propyl)- and aminopropyl-functionalized silica (Au/SiO_2_-C_3_) and [C_12_]_3_[PW_12_O_40_] nanoparticles for the preparation of water-in-oil Pickering emulsions [142]. The Au/SiO_2_-C_3_ and [C_12_]_3_[PW_12_O_40_] nanoparticles, adsorbed at the water/oil interface, act as an on-site photo-assisted heater/plasmon activator and a catalyst, respectively (Figure 23). After studying the physicochemical properties, stability, and interfacial plasmonic properties of the water-in-toluene emulsions stabilized by the two nanoparticles, the authors reported the catalytic performances of the system for the oxidation of cyclooctene using hydrogen peroxide as oxidant under UV irradiation. The results show that the turnover frequency (TOF) is 188 h^−1^ under light, while it is only 88 h^−1^ for the control experiment (i.e., [C_12_]_3_[PW_12_O_40_] alone). Moreover, the epoxycyclohexane yield is almost 100% after 75 min under light (1000 mW cm^−2^), but it is only 63% with conventional heating at 47 °C, corresponding to the temperature reached under light irradiation. The reaction proceeds without a loss of activity and selectivity after five consecutive recycles. Finally, the authors extended the scope of this system to a panel of olefins of industrial interest such as cyclooctene, (*R*)-limonene, and 1-octene. In each case, the results obtained under light irradiation are higher in terms of conversion and selectivity than after heating at 47 °C.

## 4. Conclusions and Perspectives

This manuscript provides an overview of the emerging concept of colloidal tectonics illustrated by some biotic and abiotic (supra) colloidal systems and their applications in various fields. From a historical point of view, the formation of supramolecular colloidal structures using complementary tectons (molecular building blocks), called colloidal tectonics, appeared in 2018, but a few isolated examples without generalization have been observed for two decades [15]. Following this work, new research in the field has progressed at a rapid pace. From a theoretical point of view, the phenomenon of colloidal tectonics is a limiting case between the assembly of small amphiphilic molecules or polymers into soft and fluidic micellar, vesicular, or lamellar structures, mainly due to hydrophobic interactions, and the crystalline state characterized by a regular and time-invariant three-dimensional periodic arrangement of molecules in space. Indeed, crystallization depends on molecular interactions in a simple way: highly heterogeneous interactions between molecules promote crystallization. Between these two worlds, a new paradigm is possible, as revealed by the assembly of proteins into rigid crystalline structures driven by a combination of H-bonds and hydrophobic and electrostatic interactions, producing structures such as lamellae, tubules, and polyhedrons.

For the past two decades, synthetic molecules have been used to reproduce these astonishing supramolecular assemblies that can range from the nanometer to the micrometer scale (i.e., from discrete colloidal to supracolloidal systems). The concept of colloidal tectonics, defined as the art and science of the supramolecular formation of (supra) colloidal structures using the packing of complementary molecular building blocks (i.e., tectons), creates an exciting environment for research at the intersection of molecular, colloidal, and crystal sciences. Interestingly, the colloidal tectonic approach can be compared to the colloidal building block approach. Indeed, these two terms are different but, at the same time, related in some way and may have an interdependent relationship. While the colloidal tectonic approach uses the packing of complementary molecular building blocks (tectons) to form supramolecular colloidal structures, the colloidal building block approach uses the packing of particles to obtain superstructures. The aggregates of colloidal building block particles, which can be defined as specific geometric structures obtained under the effect of attractive forces and/or external environmental effects, leading to an optimal packing of building block particles, are called colloidal clusters (or colloidal molecules) [143,144,145,146]. Therefore, the major difference between these two approaches is related to the nature of the building blocks: molecules for colloidal tectonics and particles for the colloidal building block approach. Therefore, colloidal tectonics involves using molecules (called tectons) with or without colloidal properties to provide a colloidal structure solely by self-assembly. However, these self-assembled particles can be used to form supracolloidal structures, where they act as colloidal building blocks (see above).

**Figure 24:**
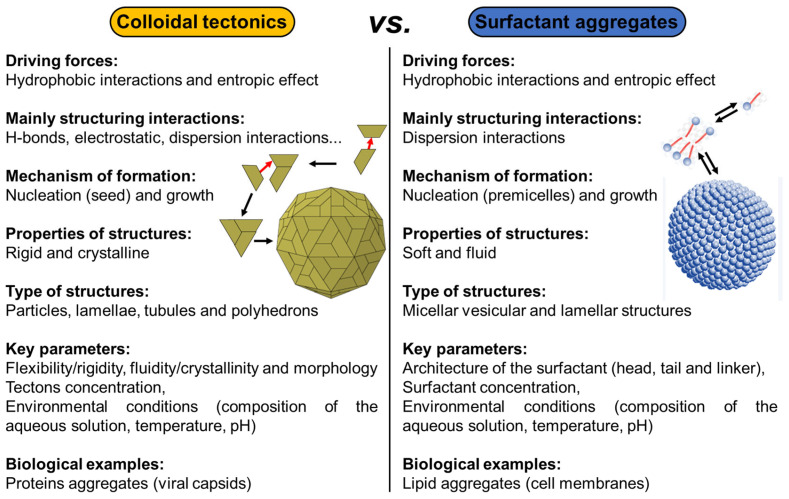
Self-assembled colloids from the colloidal tectonics approach versus surfactant aggregates.

The major advantage of the colloidal tectonic approach is that the preparation of the systems uses bottom-up self-assembly processes. However, the self-assembled structures must be stable in solvents for possible applications. Indeed, tecton–tecton interactions (or particle–particle for supracolloidal structures) must be higher than tecton–solvent interactions. Therefore, finding the right tectons is not so easy. However, in light of the scientific data illustrated in this review of the recent literature, the following empirical guidelines can be formulated: (i) colloidal architectures can be generated using either a self-complementary tecton (single-component system) or two or more complementary tectons (multi-component system); (ii) the existence of solvophobic interactions allows molecules to cluster and repel solvent molecules (e.g., for hydrophobic interactions, nonpolar molecules are held together by weak forces, such as van der Waals forces, exerted by molecular surfaces); (iii) high structural rigidity and/or well-defined crystallinity are required via strong and/or directional attraction (H-bonds, electrostatic interactions, etc.), leading to the growth of the assemblies; and (iv) steric and/or electrostatic repulsions are needed to inhibit growth at the colloidal scale. Thus, tectons can readily assemble into rigid and crystalline (or pseudo-crystalline) structures driven by a combination of hydrophobic, H-bonding, and electrostatic interactions, whereas the assembly of surfactants forms dynamic, soft, and fluidic objects mainly driven by hydrophobic interactions (Figure 24).

These two opposing behaviors are due to the existence of complementary interactions (i.e., H-bonds and/or electrostatic interactions). The self-assembled structures obtained from the colloidal tectonics approach resemble the assembly of proteins such as viral capsids as opposed to the traditional self-assembly of amphiphilic molecules that are fluid and flexible. Consequently, colloidal tectonics opens the way to new protein-mimetic materials. However, while surfactant morphology is a key parameter to guide surfactant assembly (lipid mimicry), the control of intermolecular interactions, structural flexibility/rigidity, and fluidity/crystallinity are essential to successfully mimic the morphologies of protein assemblies. On the other hand, it is commonly accepted that micellization is a two-step process: (i) surfactant molecules in the solution start to associate into small aggregates at a concentration below the CMC, and (ii) they grow to form larger aggregates (e.g., micelles at the CMC) as the concentration increases. In a similar way, self-assembly by colloidal tectonics can be interpreted in terms of nucleation/growth: (i) the subunits begin by forming a seed under the action of a local concentration fluctuation (nucleation), and (ii) other subunits that remain free then bind sequentially to this seed to make it grow rapidly until the formation of the aggregate. However, the experimental monitoring of the assembly processes of these objects remains a challenging task, as it requires probing both nanometric spatial scales and time scales ranging from microseconds to hours. Investigations are therefore needed to explain the mechanisms. For example, static light scattering, time-resolved X-ray scattering, and numerical computation techniques (Monte Carlo and molecular dynamics simulations) will undoubtedly contribute to the understanding of the mechanisms at the molecular scale by providing the structure of the most stable transient species without presupposing their nature.

The common goal is to control molecular self-assembly to produce colloidal systems with predetermined properties and/or functions in order to apply them in various fields. Current or planned applications cover catalysis, the controlled release of drugs or nucleic acids, and cosmetics. However, other applications are possible, such as obtaining molecular machines, artificial viruses for medicine, intelligent and environmentally adaptable objects, etc. Indeed, the colloidal tectonics approach can be used for the development of new smart materials because large structures are easily accessible using a bottom-up approach as they are composed of small molecules, requiring fewer synthesis steps. This approach is therefore highly multi- and interdisciplinary because the scope of this research goes far beyond the traditional boundaries of chemistry. Furthermore, it should be noted that the colloidal tectonics approach is an environmentally friendly process since it uses “green” syntheses by supramolecular assemblies (e.g., energy efficiency, minimization of synthesis steps, minimization of hazardous solvents, and possible use of bio-sourced materials). However, from an economic point of view, the cost and economic viability of the colloidal tectonic approach depend on the system (nature of the tectons, their prices, and the required quantity) and also on the intended applications. For example, the use of native CDs as tectons seems to be very interesting because they come from renewable bio-based raw materials and are also highly biocompatible, environmentally friendly, and not harmful to human health. However, considering the cost, even if the industrial production of native CDs is a relatively low-cost production, the formation of stable emulsions by colloidal tectonics (see above) requires a non-negligible amount of CDs (10 wt. %), which remains a significant limiting factor compared to current formulations stabilized by molecular surfactants. However, the value of a product depends on its composition, formulation, and application, i.e., the finished products show considerable variability in their value. Indeed, high-value-added products are intended for personal care and pharmaceuticals. Medium-value products are intended for the food and animal, chemical, and materials industries, while low-value-added products are available in larger quantities and are used in the energy and bioremediation sectors. Since the three sectors where producers can obtain higher market prices are cosmetics, healthcare, and food additives, the use of the colloidal tectonic approach is currently limited to high- and medium-value products. However, the growing demand for environmentally friendly products and processes could change this.

With the number of supramolecular colloidal systems being limited only by our imagination, colloidal tectonics, through its power over the expression of matter, invites creativity and innovation to solve current and future scientific challenges. Chemistry has expanded from molecular chemistry to supramolecular chemistry and is now evolving towards adaptive chemistry through constitutional dynamic chemistry. Indeed, the adaptation of colloidal assemblies, in response to external agents, switching processes, and morphological or shape changes, opens the way to intelligent and evolving systems with multiple potential applications. From an academic and industrial point of view, colloidal tectonics constitutes an almost infinite playground for the systems of tomorrow, thus contributing to limitless inventiveness. Like a construction game for children, the motto of colloidal tectonics is without a doubt Build and Play!
